# Precise genotyping of circular mobile elements from metagenomic data uncovers human-associated plasmids with recent common ancestors

**DOI:** 10.1101/gr.275894.121

**Published:** 2022-05

**Authors:** Nitan Shalon, David A. Relman, Eitan Yaffe

**Affiliations:** 1 Infectious Diseases Section, Veteran Affairs Palo Alto Health Care System, Palo Alto, California 94304, USA;; 2 Washington University in St. Louis, St. Louis, Missouri 63130, USA;; 3 Department of Medicine, Stanford University School of Medicine, Stanford, California 94305, USA;; 4 Department of Microbiology and Immunology, Stanford University School of Medicine, Stanford, California 94305, USA

## Abstract

Mobile genetic elements with circular genomes play a key role in the evolution of microbial communities. Their circular genomes correspond to circular walks in metagenome graphs, and yet, assemblies derived from natural microbial communities produce graphs riddled with spurious cycles, complicating the accurate reconstruction of circular genomes. We present DomCycle, an algorithm that reconstructs likely circular genomes based on the identification of so-called “dominant” graph cycles. In the implementation, we leverage paired reads to bridge assembly gaps and scrutinize cycles through a nucleotide-level analysis, making the approach robust to misassembly artifacts. We validated the approach using simulated and real sequencing data. Application of DomCycle to 32 publicly available DNA shotgun sequence data sets from diverse natural environments led to the reconstruction of hundreds of circular mobile genomes. Clustering revealed 20 highly prevalent and cryptic plasmids that have clonal population structures with recent common ancestors. This method facilitates the study of microbial communities that evolve through horizontal gene transfer.

Horizontal gene transfer (HGT) is a major driver of microbial evolution (Soucy et al. 2015). HGT supports the rapid adaptation of microbes to ecological niches (Wiedenbeck and Cohan 2011; Polz et al. 2013) and facilitates the spread of virulence factors and antimicrobial-resistance determinants within and between microbial species (Maiques et al. 2006; von Wintersdorff et al. 2016; Deng et al. 2019). Extrachromosomal circular mobile genetic elements (ecMGEs), such as plasmids and phage with circular genomes, are of particular interest as common agents of HGT (Frost et al. 2005). Experimental methods have been developed to genotype ecMGEs in complex microbial communities, including the physical enrichment of plasmids (Sentchilo et al. 2013) and of viral particles (Shkoporov et al. 2018), and the removal of linear DNA followed by multiple displacement amplification (Jørgensen et al. 2014). In the last decade, MGE characterization has been shifting toward the use of direct shotgun sequencing of complex communities, owing to reduced cost and benchwork simplicity. Several tools scan shotgun metagenomic assemblies to identify MGE-associated sequences by searching for genetic signatures of plasmids and phage (Zhou and Xu 2010; Lanza et al. 2014; Carattoli et al. 2014; Roux et al. 2015; Roosaare et al. 2018; Robertson and Nash 2018). However, these tools do not reconstruct complete genomes and can conflate extrachromosomal with integrated forms of mobile elements, confounding the study of ecMGEs in natural settings.

A powerful approach to recover complete genomes of ecMGEs is based on metagenomic assembly graphs. In these graphs, vertices represent contigs (partially assembled DNA sequences), and edges represent contig–contig adjacencies supported by crossing reads. One formulation of the problem at hand is the identification of circular graph walks that correspond to underlying circular genomes. Yet some circular walks are in fact “phantoms,” for which the corresponding circular genomes are not present in the biological sample. These phantom walks are a result of identical (or nearly identical) DNA sequences appearing in different genomic contexts. Repeat elements such as transposons and integrated phage are common in microbial genomes. Moreover, natural microbial communities have been shown to simultaneously harbor closely related ecMGEs that differ by only a few genome rearrangements (Conlan et al. 2014; He et al. 2015; Suzuki et al. 2019). Repeats and rearrangements produce complex graphs riddled with phantom walks, complicating the task.

Several tools traverse the assembly graph in an attempt to reconstruct complete genomes of ecMGEs (Rozov et al. 2017; Antipov et al. 2019; Pellow et al. 2021). These tools efficiently search for circular graph walks that may correspond to ecMGEs, yet their heuristic nature makes them highly susceptible to errors. A review estimated that >25% of ecMGE genomes reported by existing approaches do not correspond to true ecMGEs (Arredondo-Alonso et al. 2017). Meanwhile, theory has been developed to formulate the correctness of walks in assembly graphs (Obscura Acosta et al. 2018). However, theory revolving around the correctness of circular walks needed for the recovery of circular genomes is lacking. The recovery of MGE genomes will allow the field to conduct high-throughput computational surveys of mobile elements, strengthening the understanding of the evolution and spread of mobile elements and their genetic cargo in natural environments.

The goal of this work was to develop a tool (DomCycle) that reliably infers circular genomes of ecMGEs from short-read shotgun DNA data derived from complex microbial communities. A second goal was to validate DomCycle using negative controls without circular genomes, reference ecMGE data, and realistic simulations of evolving mobile elements. A final goal was to showcase the discovery potential of DomCycle by applying the tool to metagenomic data generated from diverse microbial communities.

## Results

**Figure 1.**
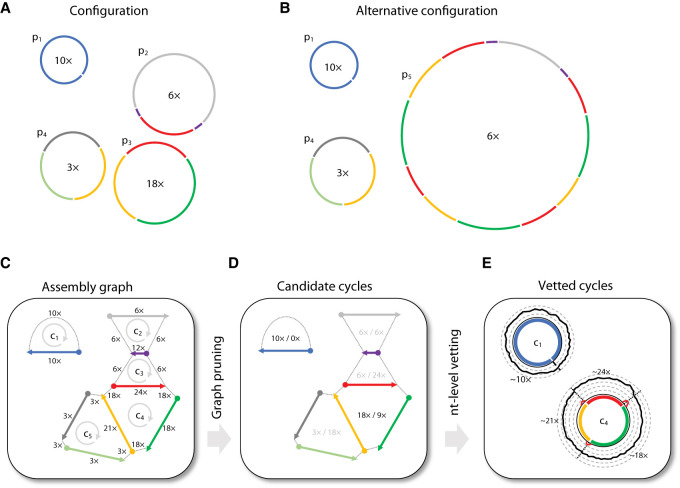
Approach overview. (*A*) A manufactured example of a latent genome configuration and assembly with eight unique contigs. Contigs are color-coded, and four DNA genomes are marked p_1_–p_4_ with their x-coverage specified in their center. For example, p_2_ has an x-coverage of 6× and contains only three unique contigs, as the short purple contig appears twice. The corresponding assembly graph is shown in *C*. Under this configuration, cycle c_4_ is a real cycle that corresponds to p_3_. (*B*) An alternative genome configuration that produces the same graph. The presence of the complex genome p_5_, which corresponds to an involved circuit in the graph, affects the multiplicity of all visited cycles. For example, the multiplicity of cycle c_4_ is equal to four because the circuit that corresponds to p_5_ visits the red contig four times while traversing in a loop along the contigs of c_4_. Note that under this configuration, cycle c_4_ is a phantom cycle. (*C*) The assembly graph that corresponds to the configurations shown in *A* and *B*. Directed edges appear within a single contig (going from the 5′ end to the 3′ end, represented with arrows) and between adjacent contigs (going from the 3′ end to the 5′ end, represented with dotted lines). The graph contains five cycles (c_1_–c_5_), and the x-coverage of edges is indicated. (*D*) The cycle score *σ*_*c*_/*τ*_*c*_ is specified in the center of each cycle for the graph shown in *C*. The algorithm recovers all candidate dominant cycles (*σ*_*c*_/*τ*_*c*_ > 1; score colored black) and discards nondominant cycles (score colored gray). For example, cycle c_4_ has a score of two (*σ*_*c*_ = 18 × , *τ*_*c*_ = 9 × ), making it a candidate dominant cycle. (*E*) In our implementation, nucleotide-level read profiles are computed for all candidate dominant cycles using all paired reads for which at least one side mapped to the cycle, and the algorithm returns dominant cycles with estimates of x-coverage. Reads are grouped into cycle-supporting reads (black line) and nonsupporting reads (red line). For example, the x-coverage of supporting reads along c_4_ varies, with three short stretches of nonsupporting reads on contig–contig seams. Average read x-coverage values for portions of the cycles are shown on the plot.

We developed a theoretical framework and associated tool (DomCycle) to recover near-complete genomes of ecMGEs from metagenome assembly graphs (Fig. 1). In the assembly graph, *circuits* are circular walks that correspond to circular chains of contigs. Although every circular genome produces a corresponding circuit in the graph, inferring the underlying genomes from the graph is nontrivial, as different underlying genome configurations can result in the same graph. This is illustrated by two manufactured configurations (Fig. 1A,B) and the graph that corresponds to both (Fig. 1C). To simplify the problem, we focused on *cycles*, which are circuits that do not include any contig more than once. Yet, even cycles may have no true corresponding genome. Repeat elements can result in spurious cycles (called here “phantom” cycles) for which no corresponding circular genome exists in one or more of the possible latent genome configurations (illustrated in Fig. 1C). Our goal was to develop a tool that calculates robust mathematical attributes of cycles that can help distinguish between phantom and real cycles.

### Algorithm that recovers all dominant graph cycles

Our approach is based on a new concept called “dominant cycles.” Dominant cycles are loosely related to the previously introduced notion of “dominant plasmids” (Antipov et al. 2019), yet they are grounded in graph theory. We assume a latent configuration of circular genomes that produces an assembly graph (see Methods). For every cycle *c* in the graph, the *bottleneck coverage σ*_*c*_ is the minimal read coverage along the edges of the cycle, the *external coverage τ*_*c*_ is the total number of paired reads leading in or out of the cycle (averaging in and out), and the cycle score is the ratio *σ*_*c*_/*τ*_*c*_ (Fig. 1D). We call a cycle *dominant* if *σ*_*c*_/*τ*_*c*_ > 1.

We also define two latent variables. Our latent variable of interest is the coverage *ψ*_*c*_ of the genome associated with a cycle *c* (*ψ*_*c*_ ≥ 0 by definition and *ψ*_*c*_ = 0 if the genome is not part of the configuration). A second latent variable is the multiplicity *μ*_*c*_, which is equal to the number of times the contigs of the cycle appear consecutively within the context of a larger circuit, such as within a tandem repeat (defined in the Methods). For clarity, we emphasize that the last and possibly incomplete pass along the contigs of the cycle is counted toward the multiplicity, as shown in Figure 1B. Our main theoretical result is the bound *σ*_*c*_ − *μ*_*c*_ × *τ*_*c*_ ≤ *ψ*_*c*_. This lower bound on *ψ*_*c*_ means that either any dominant cycle is real (i.e., *ψ*_*c*_ > 0) or it has a multiplicity greater than or equal to the cycle score (i.e., *σ*_*c*_/*τ*_*c*_ ≤ *μ*_*c*_). Although high-multiplicity cycles are theoretically possible, they require complex tandem structures as shown in Figure 1B, and it is not clear how common they are in microbial genomes. To gauge the prevalence of high-multiplicity cycles that are dominant and phantom (called dominant-phantom), we used empirical data, presented below.

We developed an algorithm that recovers all dominant cycles in an assembly graph. In the implementation of the algorithm, contig–contig edges in the assembly graph are inferred using paired reads while bridging possible small gaps (for details, see Methods). Candidate cycles are vetted on a nucleotide-level basis by comparing the ratio between the number of supporting reads, for which both sides map with proper orientations to the cycle, and the number of nonsupporting reads (Fig. 1E). This makes the approach robust to common forms of misassembly. Stochasticity in read coverage is modeled using a binomial distribution to identify vetted dominant cycles with scores that are significantly larger than one. We note that DNA replication (Antipov et al. 2016) and sequencing biases (Sato et al. 2019; Browne et al. 2020) introduce systematic biases to read coverage, which poses a challenge to our assumption of uniform coverage. The algorithm yields near-complete circular genomes (not necessarily complete owing to possible small gaps between consecutive contigs) that correspond to all vetted dominant cycles.

### Validation using reference genomic data

**Figure 2.**
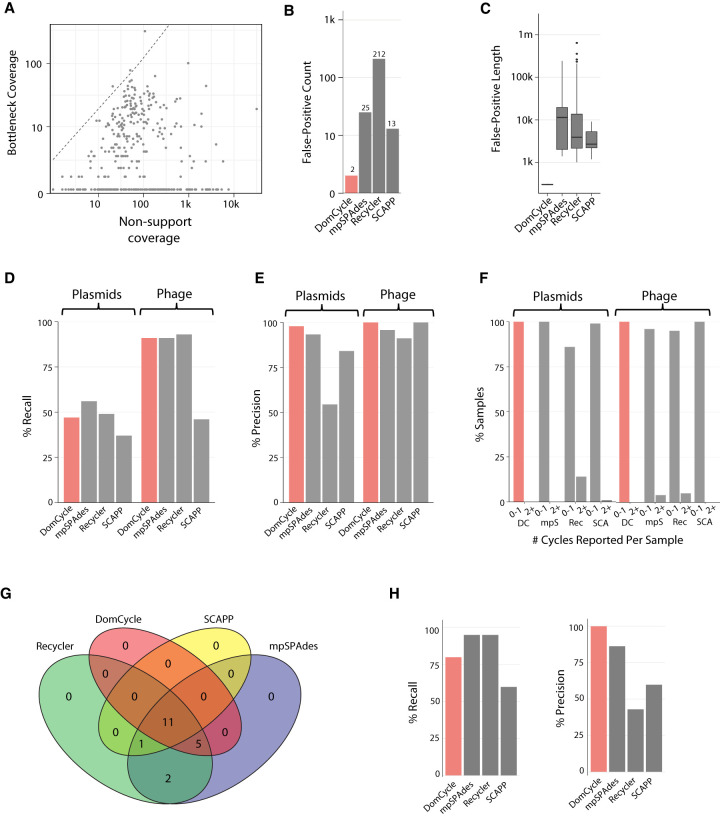
Specificity and sensitivity estimated with reference sequences. (*A*) The distribution of the bottleneck coverage versus the total nonsupport coverage among cycles inspected by DomCycle on a metagenomic data set simulated on 155 reference chromosomal sequences. The dashed line shows the threshold of significance (*P* < 0.01) for the global nucleotide test. Three cycles had global scores significantly above one and were selected as candidate dominant cycles. (*B*) The number of phantom cycles reported on the simulated metagenomic data set, comparing the performance of DomCycle, metaplasmidSPAdes (denoted by mpSpades), Recycler, and SCAPP. Two candidate dominant cycles passed the local nucleotide test. (*C*) The length distribution for phantom cycles reported on the simulated metagenomic data set. The two phantom cycles reported by DomCycle are <1 kb. (*D*) Two hundred simulated data sets were generated from 100 reference plasmids and 100 reference phages. Shown are recall estimates for DomCycle, metaplasmidSPAdes, Recycler, and SCAPP. Recall was defined as the number of runs with one or more correctly reported cycles divided by the total number of runs. (*E*) Precision estimates for DomCycle, metaplasmidSPAdes, Recycler, and SCAPP for the same data sets as in *D*. Precision was defined as the number of correctly reported cycles divided by the total number of reported cycles. (*F*) The number of reported cycles per sample for DomCycle, metaplasmidSPAdes, Recycler, and SCAPP when tested on reference plasmids and phages. DomCycle reported, at most, a single cycle in all cases. (*G*) Breakdown of the elements reported on the CAMI Low data set. (*H*) Recall and precision on the CAMI Low data set.

We tested the empirical prevalence of dominant-phantom cycles using simulated data derived from bacterial chromosomal genomes. DomCycle was applied to simulated shotgun data derived from an in silico mixture of 155 linearized genomes, including several conspecific strains (total assembly size 450 Mbp) (species detailed in Supplemental Table 1). By design, the generated assembly graph contained only phantom cycles because genomic DNA was linearized and no ecMGEs were included. DomCycle reported only two dominant-phantom cycles, which were both short (294 bp and 315 bp) and with relatively low scores (2.4 and 1.3, respectively) (Fig. 2A). We applied Recycler (Rozov et al. 2017), metaplasmidSPAdes (Antipov et al. 2019), and SCAPP (Pellow et al. 2021) to the same data set. These tools reported 13–212 phantom genomes (Fig. 2B), with a median element length of 2.7–11.4 kb (Fig. 2C). The analysis of tandem repeats, which are genetic constructs known to complicate assembly graphs, partially explained why DomCycle outperforms Recycler and metaplasmidSPAdes in terms of precision (Supplemental Fig. S1). DomCycle completed work in this data set in 7 h, displaying similar performance as existing tools (Supplemental Fig. S2).

We evaluated the performance of DomCycle on 100 reference plasmids and 100 reference phage genomes, all assayed individually (Supplemental Table 2). Recall was 47% for plasmids and 91% for phages, slightly lower than the maximal performance of the other tools (Fig. 2D). DomCycle had near-perfect precision (Fig. 2E) and consistently reported only a single cycle (or no cycle at all), whereas all other tools occasionally split a single genome into multiple reported elements (Fig. 2F). Recall and precision were robust to changes in key thresholds, such as the score cutoff that defines dominant cycles (Supplemental Fig. S3). We compared all tools on a data set derived from a real microbial community (CAMI) (Sczyrba et al. 2017) that included 40 genomes and 20 ecMGEs (Fig. 2G). Again, DomCycle was precise at the expense of sensitivity (Fig. 2H). To summarize, DomCycle effectively limits reporting phantom cycles while achieving recall values that are only slightly inferior to existing tools.

### Validation using simulated mobile element data

**Figure 3.**
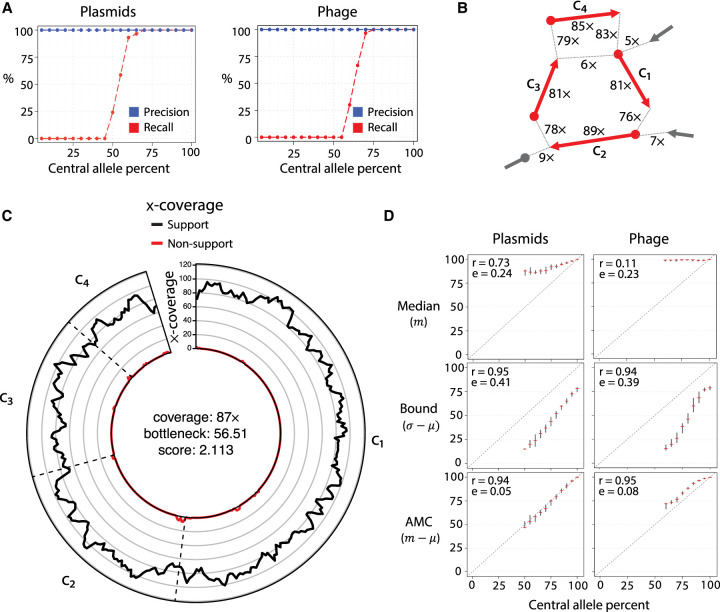
DomCycle performance on recombining plasmids and partially induced phage. (*A*) The recall (red) and precision (blue) for simulations at varying central allele frequencies. Each point represents the results of 30 trials at a single central allele frequency. Central allele frequency was calculated as the percentage of the total x-coverage contributed by the central allele. (*B*) Example of a recovered central allele plasmid with a frequency of 55%. Shown is a subset of the assembly graph focusing on a single reported cycle. The graph is represented as in Figure 1C. The recovered cycle is colored in red, and adjacent graph edges that are not part of the cycle are colored in gray. Coverage units show the edge coverage, *W*(*e*). Labels on internal edges show contig names. (*C*) The nucleotide-level cycle coverage profile corresponding to the cycle depicted in panel *B*. The coverage of cycle supporting reads is colored in black, and the coverage of nonsupporting reads is colored in red. (*D*) Median coverage, lower bound coverage, and adjusted median coverage (AMC) as predictors of true allele frequency. The Pearson correlation coefficient (denoted with “r”) and root mean squared deviation (denoted with “e”) are shown for each predictor. In each small cross, the horizontal line shows the median metric value of an estimator at a given central allele frequency, and the vertical line depicts the interquartile range of the estimator, created with 30 replicates for each allele frequency. Diagonal dotted lines show the true central allele frequency.

We simulated two realistic scenarios of evolving mobile genetic elements. The first scenario involved a single plasmid (central allele) and several variant plasmids (variant alleles) that were individually distinguished from the major allele by a single-genome rearrangement event (insertion, deletion, or inversion). The second scenario simulated a semi-induced prophage. It involved a phage that both appeared in a circular form (central allele) and integrated several times into a linear genome (variant alleles). For both scenarios, we tested recall and precision as a function of the frequency of the central allele (Fig. 3A). Central alleles were recovered when the allele frequency surpassed 45% for plasmids and 55% for phages. Despite a background of convoluted genome rearrangements, the precision was perfect in all cases; that is, all cycles reported by DomCycle were associated with real underlying genomes, and multiple cycles were never reported. To illustrate the performance of DomCycle, we show the underlying graph cycle (Fig. 3B) and the distribution of mapped reads along the cycle (Fig. 3C) for a single successful plasmid run. In comparison, Recycler and metaplasmidSPAdes had some precision issues, with Recycler showing a minimum precision of 0% and metaplasmidSPAdes achieving <90% precision in some cases (Supplemental Fig. S4).

We leveraged our knowledge of the true underlying coverages to examine the performance of three estimators of genome coverage (Fig. 3D). The best estimator was the adjusted median coverage (AMC), defined as *x*_*c*_ = *m*_*c*_ − *τ*_*c*_, where *m*_*c*_ is the median coverage along the cycle. AMC was both highly correlated with true coverage values (Pearson's coefficient *ρ* = 0.94) and had a root-mean-square deviation (RMSD) of 0.05 and 0.08 for plasmids and phages, respectively. In summary, the analysis of simulated data showed the ability of DomCycle to recover dominant cycles and accurately predict their coverage in a scenario with evolving mobile elements.

### Circular genetic elements in the human gut

**Figure 4.**
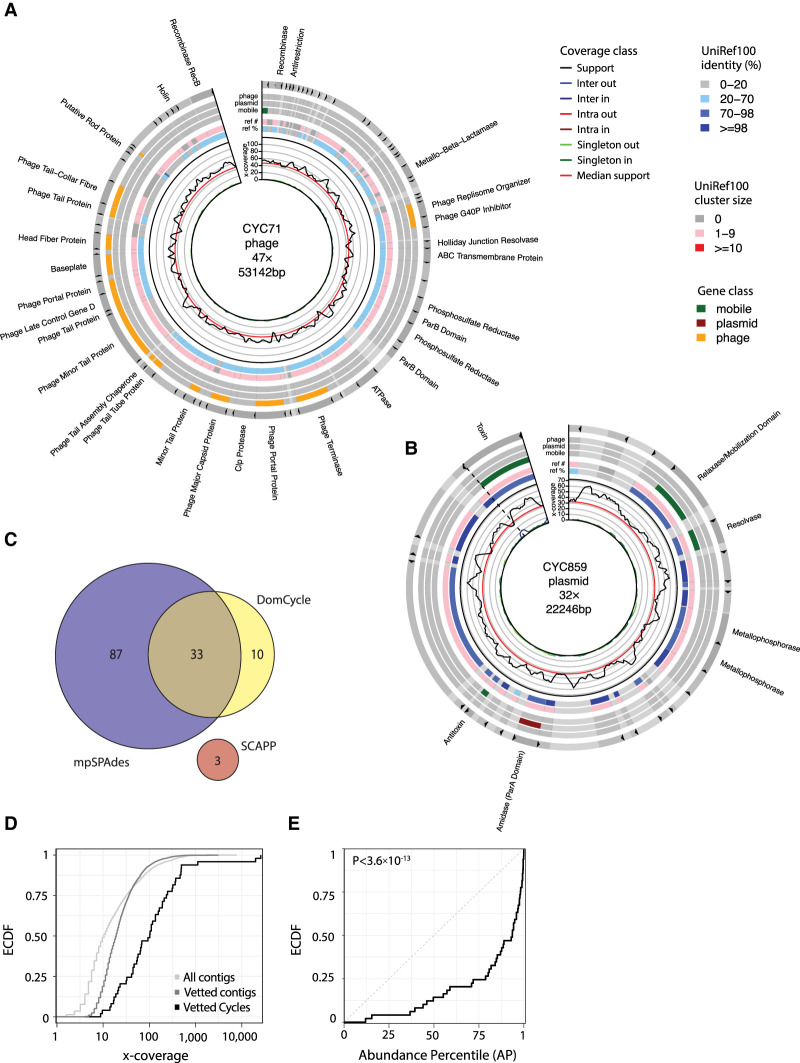
ecMGEs in the gut of a healthy adult. (*A*) A putative gut phage. Shown from *inner* to *outer* circles are nucleotide-level coverage profiles, UniRef100 hits (sequence identity [AAI] to the best UniRef100 hit and the number of UniProt genes in the UniRef100 cluster), and gene classification. Gene descriptions for select phage-associated genes are specified *outside*. Cycles are cut open at the start of their linear sequence for visualization purposes. (*B*) Same as *A*, for a putative plasmid. (*C*) Genomes corresponding to reported cycles pairwise aligned and clustered using a threshold ANI of 95%. Shown is cluster breakdown for DomCycle, metaplasmidSPAdes, and SCAPP. (*D*) Shown are the empirical cumulative distribution functions (ECDFs) for the AMC of the 49 vetted dominant cycles that correspond to putative ecMGEs (black), the background AMC defined as the AMC of contigs vetted in the same way as dominant cycles except for the circularity condition (dark gray), and the background coverage all contigs in the assembly (light gray). (*E*) Shown for all 49 vetted dominant cycles is the ECDF of the cycle abundance percentile, defined as the percentile of the cycle AMC within the background AMC distribution. *P*-value computed using a one-sided Kolmogorov–Smirnov test is shown.

We applied DomCycle to metagenomic shotgun data composed of 200 million paired reads derived from stool of a healthy adult (Yaffe and Relman 2020). The contig-level and nucleotide-level assessment of cycles was in general agreement (Supplemental Fig. S5), with only eight cycles that were dropped owing to local score filtering and abnormal read coverage that likely stemmed from assembly artifacts (Supplemental Fig. S6). After stringent cycle vetting, 49 dominant cycles and their corresponding ecMGEs remained. The MEGAHIT assembler (our choice of assembler) and the metaSPAdes assembler (used by metaplasmidSPAdes) agreed on 36 (67%) of the reported cycles (Supplemental Fig. S7). The lengths of the 49 ecMGE genomes ranged from 0.6 kb to 185 kb (median length 4.8 kb). In terms of complexity, 68% of the ecMGE genomes were associated with a self-loop formed by a single contig, 13% were self-loops that included a small assembly gap that was bridged using paired reads, and the remaining 19% of genomes spanned multiple contigs. We offer an example of a putative phage (Fig. 4A) and a putative plasmid (Fig. 4B; all ecMGEs are shown in Supplemental Fig. S8). Of note, among the recovered ecMGEs was a complete genome of a member of the crAssphage family (Dutilh et al. 2014), a group of prevalent *Bacteroides* phage (Supplemental Fig. S9).

We applied metaplasmidSPAdes and SCAPP to the same gut data set (Recycler did not complete the analysis within a week and was excluded). DomCycle and metaplasmidSPAdes were generally in agreement, whereas SCAPP reported only three ecMGEs that clustered separately (Fig. 4C). The number of ecMGEs identified by metaplasmidSPAdes and SCAPP was similar to their expected rate of false-positives (computed based on Fig. 2B), suggesting that the low precision of current tools confounds the analysis of real data (Supplemental Fig. S10). Furthermore, cycles reported only by metaplasmidSPAdes had low scores that were close to one, supporting the possibility that many of them are in fact phantom cycles (Supplemental Fig. S11).

The AMC of the 49 ecMGEs ranged from 8.8× to 25,960× and was on average 10-fold higher than the average coverage of all contigs in the assembly (Fig. 4D). This suggested that the detected ecMGEs have elevated abundance levels compared with the average abundance of bacterial members in the microbial community. However, to properly interpret AMC values, one needs to take into account the reduced probability of detecting low-coverage cycles owing to the stringent cycle vetting procedure. We computed for each ecMGE its *abundance percentile* (AP), defined as the percentile of its AMC score within a background distribution of AMC values estimated using all contigs in the assembly (Methods). Even after this normalization, ecMGEs were significantly abundant within the community (Kolmogorov–Smirnov test *D* = 0.543, *P* < 3.6 × 10^−13^), with 20 ecMGEs (41%) above the 95th AP (Fig. 4E). Analysis of shotgun data (96 million paired reads) derived from the stool of a second healthy individual (Yaffe and Relman 2020) qualitatively recapitulated the elevated abundances of ecMGEs (Supplemental Fig. S12).

### Comparing DomCycle to existing tools

Recycler, the first tool that identified ecMGEs from assembly graphs, uses a heuristic approach that results in limited precision when applied to reference ecMGEs (Figs. 2E,H) and to realistic evolutionary scenarios (Supplemental Fig. S4). SCAPP, designed to address the precision issues of Recycler by using genetic signatures of mobile elements, has improved precision at the expense of sensitivity. Despite SCAPP's improved precision owing to its reference-based approach, SCAPP may still report complex linearly inserted elements with mobile genes as demonstrated in Figure 2, B and C.

### Circular mobile elements are abundant in diverse environments

**Figure 5.**
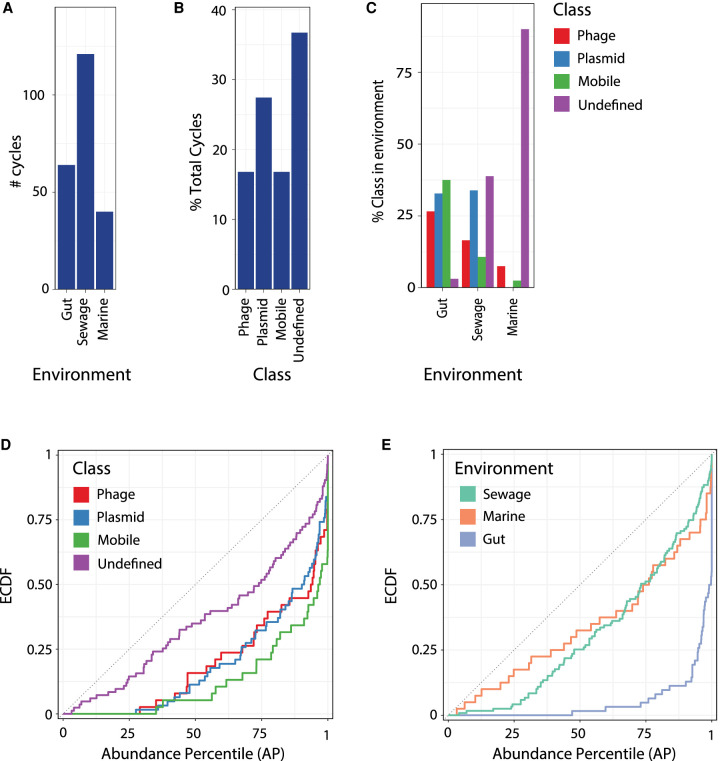
ecMGEs in the human gut, sewage wastewater, and marine environments. (*A*) The number of ecMGEs identified in each environment. (*B*) The percentage of ecMGEs with assigned functional classes. (*C*) The percentage of ecMGEs within each environment, stratified by class. (*D*) The ECDF of abundance percentile values of ecMGEs, stratified by class. (*E*) Same plot as in *D*, stratified by environment.

We applied DomCycle to 30 environmental shotgun libraries, including human stool (median 104 million reads per sample) (The Human Microbiome Project Consortium 2012), sewage wastewater (median 48 million reads per sample) (Hendriksen et al. 2019), and the marine environment (median 37 million reads per sample; 10 samples each) (Supplemental Table 3; Biller et al. 2018). In total, we identified 720 dominant cycles and reconstructed their associated ecMGE genomes. Analysis was limited to 221 ecMGEs (29%) that were at least 1 kb long (Fig. 5A). All ecMGEs were classified based on annotations of predicted genes, as putative plasmids (28%), putative phage (17%), unspecified mobile elements (17%), and undefined elements (38%) (Fig. 5B). The three environments differed in the distribution of classes (chi-squared test *P* < 10^−16^), with the gut relatively depleted for undefined elements, sewage enriched for plasmids and undefined elements, and marine samples enriched for undefined elements (Fig. 5C). Recapitulating our observation in the two gut samples of Figure 4, the 221 recovered ecMGEs were highly abundant (KS-test *P* < 10^−16^), with 73% of ecMGEs above the 95th AP within their respective communities. All ecMGE classes were associated to some degree with elevated abundance levels (Fig. 5D). Elevated abundance of ecMGEs was observed in all environments yet was most prominent in the human gut (Fig. 5E). The high abundance of ecMGEs we observed may be explained by high copy numbers, preferential targeting of abundant microbial hosts, and/or promiscuous ecMGE-host interactions.

### Highly prevalent plasmids are rapidly circulating

**Figure 6.**
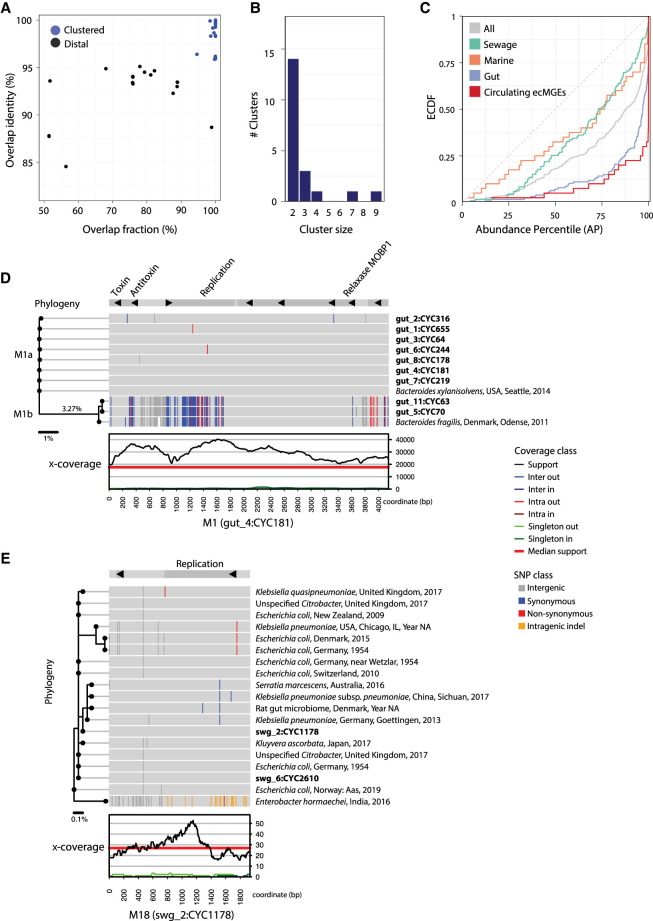
Analysis of circulating ecMGEs. (*A*) The distribution of cycle tightness metrics across pairs of ecMGEs (>1 kb) in the 32 samples assayed. The overlap identity is defined as the average identity of aligned regions between two cycle genomes. The overlap fraction is defined as the percentage of two cycle genomes that align. For a cycle pair, genomic similarity is defined as the overlap identity times the overlap fraction. Cycle pairs are linked if their genomic similarity is >0.95, and clusters are defined as groups of linked cycles. (*B*) The distribution of the number of members in clusters. (*C*) The ECDF of abundance percentile values of circulating ecMGEs compared with ecMGEs stratified by their environment and with all ecMGEs. (*D*) Detailed view of cluster M1. Data are projected onto a reference “pivot” cluster member (gut4, cycle 181) that is shown in a linear format for visualization purposes. Shown from the *bottom* up are the x-coverage profiles of the pivot ecMGE member, SNP patterns of cluster members (in bold) and reference sequences (isolate source, location, and year of collection), and annotated genes (on *top*). SNP patterns are colored according to differences from the pivot, with white indicating segments that failed to align. A phylogenic tree is shown on the *left*. The units of the scale bar *under* the tree are mean nucleotide differences. Clades M1a and M1b are marked on the plot. (*E*) Detailed view of cluster M18, represented as in *D*.

We were curious to see if we could find evidence of ecMGEs circulating within and between environments. We therefore performed pairwise genome alignments of all 286 ecMGEs (>1 kb) detected in the 32 environmental samples described in this work. A comparison of the fraction of the aligned region and the average nucleotide identity (ANI) within the aligned region suggested that ecMGEs preferentially maintain their diversity through local mutations and not through large-scale genomic changes (Fig. 6A). The ecMGEs were grouped into 244 clusters based on sequence identity using a threshold of 95% ANI (Supplemental Fig. S13). Results were robust to changes in the clustering threshold (Supplemental Fig. S14). Analysis was limited to the 20 clusters (denoted M1–M20) that had two or more members (Table 1; for cluster members, see Supplemental Table 4). Clusters were extremely tight; the average fraction of the aligned region between pairs of cluster members was 99.56%–100% (median 100%), and the average ANI within the aligned region was 98.43%–100% (median 99.9%). We refer to these clustered ecMGEs, which were reconstructed independently with minor genetic variations in multiple samples, as *circulating* ecMGEs.

**Table 1.**
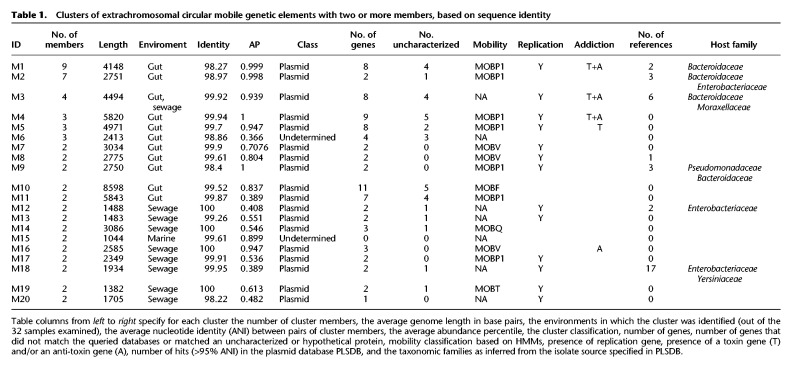
Clusters of extrachromosomal circular mobile genetic elements with two or more members, based on sequence identity

We classified 16 clusters as putative plasmids owing to the presence of mobility and/or replication genes, of which five had one or two toxin–antitoxin genes, as summarized in Table 1 (for details, see Supplemental Table 5). There were 33 uncharacterized genes in total, which made up 32% of each cluster on average (Supplemental Table 6). Clusters had two to nine ecMGE members (Fig. 6B), and all were from a single environment except cluster M3, which was observed in both gut and sewage samples. The AP of circulating ecMGEs was high (mean AP of 92%), in agreement with a commonly observed ecological association between prevalence and abundance (Fig. 6C).

Leveraging a comprehensive plasmid reference database (PLSDB, with over 26 k plasmids) (Galata et al. 2019), we identified seven clusters with near-identical reference hits (99.7% ANI on average) (Supplemental Table 7). This independent reconstruction of the circulating ecMGEs provided validation of our metagenomic approach. Moreover, the PLSDB metadata informed us about the putative host-range and environments of some of our clusters. These data suggested that our circulating ecMGEs may have a broad host-range, with five (out of six with host data) associated with multiple taxonomic families (Table 1). We discovered that the top three plasmids (M1–M3) were reported as cryptic *Bacteroides* plasmids in the 1980s (Wallace et al. 1981; Sóki et al. 2010). On the other hand, the 13 circulating ecMGEs that did not have a close reference in PLSDB (>95% ANI) despite their prevalence in the general population highlight the discovery power of our metagenomic approach.

We proceeded to augment clusters with reference genomes (where available) and inferred cluster-specific phylogenic trees. The most prevalent plasmid (M1; 4138 ± 10 bp long) was observed in nine gut samples and had two isolate-based reference genomes (Fig. 6D). M1 is composed of a major clade (M1a; isolated previously from *Bacteroides xylanisolvens*) and a minor clade (M1b; isolated previously from *Bacteroides fragilis*), with a genetic distance of 3.27% separating the two inferred clade ancestors. Both have shallow clonal trees distinguished by a handful of SNPs, with a mean ANI of 99.95% and 99.76% between clade members for M1a and M1b, respectively. Out of the 12 gut samples we assayed, M1a was present in seven out of 12 (58 ± 22%), making it one of the most prevalent plasmids recorded in the human gut to date. A coalescence analysis suggests M1a has gone through a clonal expansion merely ∼600–1200 yr ago (Supplemental Note 4).

Another cluster of interest is M18, a short cryptic plasmid (1934 bp) recovered from two sewage metagenomic samples and independently reconstructed from 17 isolates (primarily *Enterobacteriaceae* species) that were collected across the globe (Fig. 6E). The clonal population structure of M18 is in discordance with its host species, suggesting it is freely circulating between a diverse set of hosts.

The remaining five ecMGE clusters that have reference genomes suggest that ecMGEs are found in diverse environments and bacterial hosts (Supplemental Fig. S15). The nine ecMGE clusters with sufficient members to estimate their phylogeny had clonal populations with only 0%–3.2% putative recombined sites, suggesting recombination between circulating ecMGEs is relatively rare (Supplemental Fig. S16). Finally, we noted several examples of clusters with an uneven distribution of SNPs along their genomes, suggestive of possible adaptive evolution or recombination (Supplemental Fig. S17).

## Discussion

We present an algorithm that recovers all dominant cycles in a metagenomic assembly graph and reconstructs their corresponding genomes. Our implementation achieves high precision by combining graph theory and nucleotide-level vetting of cycles. We show that in the context of microbial communities, dominant cycles likely correspond to true extrachromosomal circular DNA. Application to complex evolutionary scenarios and reference data reliably recovers ecMGEs with a negligible false-positive rate.

The major limitation of the approach is that it recovers only circular genomes that correspond to dominant cycles. In reality, some mobile elements are linear, and circular mobile elements can contain repeat elements that produce circuits, both of which would not be detected by our approach. Although linear extrachromosomal DNA is outside the scope of this work, the ongoing transition to long-read sequencing technologies will transform some circuits to cycles. With meticulous handling of environmental samples, long reads can extend up to 5–10 kb (Suzuki et al. 2019; Moss et al. 2020). Combining long reads with the approach presented here will allow genotyping of complex ecMGEs, such as MGEs that contain insertion sequences (typically < 2.5 kb) and short transposons. Longer repeat elements and complex rearrangements that require bridging over 10 kb or more can be addressed with additional experimental work, such as Hi-C (Beitel et al. 2014; Stalder et al. 2019; Yaffe and Relman 2020; Cockram et al. 2021), or by sampling the same community multiple times (Nissen et al. 2021; MetaHIT Consortium et al. 2014). Another weakness is the requirement that for cycles to be identified, they must be dominant. A family of partially overlapping circular genomes can result in nondominant cycles and therefore be overlooked entirely (as shown in Fig. 3A, where the frequency is roughly <50%). Although it is not clear to what extent this phenomenon is common in natural communities, the component-based approach taken by metaplasmidSPAdes can be more appropriate in this case, as the entire family can produce a single identifiable graph component.

The approaches of metaplasmidSPAdes and DomCycle are related. MetaplasmidSPAdes works by consecutively removing graph edges (sorted by their coverage, from low to high) and reporting graph components that transiently form during the removal process. This approach will recover all dominant cycles, yet not all components reported by metaplasmidSPAdes necessarily correspond to true ecMGEs. In fact, linear genetic elements (such as tandem repeats and composite transposons) that are integrated in numerous genomic locations can form transient graph components and may get reported even though they are not circular nor extrachromosomal. To overcome this drawback, a complete accounting of the weights of the edges leading into and out of components is required. In this manner, it is possible to define and compute component scores (akin to cycle scores) in order to improve the precision of metaplasmidSPAdes. From a practical perspective, metaplasmidSPAdes introduces an unnecessary dependency on the metaSPAdes assembler.

Application of our approach to 32 environmental samples uncovered 20 clades of ecMGEs (primarily gut and sewage plasmids), showcasing the strength of metagenomic approaches in tapping into understudied environmental plasmids. The low sequence diversity and clonal population structure we report here were observed in plant-associated virulence plasmids (Weisberg et al. 2020). Plasmid clonality is particularly striking when contrasted with the population structure of their bacterial hosts, which partake in pervasive recombination at levels that can obscure strain phylogeny (Garud et al. 2019; Sakoparnig et al. 2021; Shi et al. 2022). The clonality and lack of diversity can be partially attributed to their simplified ecological niche, which may favor rapid cycles of selective sweeps. Further characterization of the adaptive landscape of MGEs in the gut and elsewhere will require a larger data set. In summary, this work presents a new tool that allows reconstruction of ecMGEs from readily available public metagenomic shotgun data and that may help to elucidate the evolution and dissemination of mobile genetic elements within and between environments.

## Methods

### Graph theory

#### Assembly graph

A *contig x* is a sequence of nucleotides from the set {*A*, *G*, *C*, *T*} and is associated with a *head vertex* $v_{x}^{head}$ (5′ contig end), a *tail vertex* $v_{x}^{tail}$ (3′ contig end), and an *internal directed edge* $e(x)=(v_{x}^{head},\;v_{x}^{tail})$. A pair of contigs, *x*, *y*, is associated with an *external directed edge* $e(x,y)=(v_{x}^{tail},v_{y}^{head})$. A contig set is called *nonredundant* if for every contig *x* in the set, the reverse complement of *x*, denoted by *x*′, is not in the set. Let *X* be a nonredundant contig set. We denote all vertices of *X* by $V=\cup_{x\in X}\{v_{x}^{head},v_{x^{\prime }}^{head},v_{x}^{tail},v_{x^{\prime }}^{tail}\}$ and all edges of *X* by $E=\cup_{x\in X}\{e(x),e(x^{\prime })\}\cup \cup_{x,y\in X}\{e(x,y),e(x^{\prime },y),e(x,y^{\prime }),e(x^{\prime },y^{\prime })\}$. The *directed assembly graph* of the contig set *X* is then defined to be *G* = (*V*, *E*).

#### Circuits and cycles

Let $\bar{x}=(x_{0},x_{1},\ldots ,x_{n-1})$ be a sequence of directed contigs in *X*, or formally, it holds that ($x_{i}\in X)$∨($({x^{\prime }}_{i}\in X)\;\mathrm{for}\;i=0,\ldots ,n-1$. The *circuit* $p(\bar{x})$ is a walk in *G* that traverses the graph through the vertices $V(\;p(\bar{x}))=(v_{x_{0}}^{head},v_{x_{0}}^{tail},v_{x_{1}}^{head},v_{x_{1}}^{tail},\ldots ,v_{x_{n-1}}^{head},v_{x_{n-1}}^{tail},v_{x_{0}}^{head})$ and with matching edges $E(\;p(\bar{x}))=(e(x_{0}),e(x_{0},x_{1}),\;e(x_{1}),\;\ldots ,e(x_{n-1},x_{0}))$. We note that the same circuit $p(\bar{x})$ can be represented in *n* alternative ways by selecting a different starting contig. The circuit $p(\bar{x})$ is called a *cycle* if it does not traverse a contig or its reverse complement more than once, or formally, max_*x*∈*X*_|{0 ≤ *i* < *n*:(*x*_*i*_ = *x*)∨(*x*_*i*_ = *x*′)}| ≤ 1. The reverse complement of $p(\bar{x})$, denoted by $p{\left(\bar{x}\right)}^{\prime }$, is the circuit associated with the contig sequence $\left({x^{\prime }}_{n-1},\;\ldots ,{x^{\prime }}_{0}\right)$.

#### Cycle and edge coverage

We denote the collection of all circuits in the graph *G* by *C*. Note that by definition there is a one-to-one relationship between *C* and all possible circular genomes composed of oriented contigs selected from *X*. The *genome coverage H*:*C* → ℝ_≥0_ is a latent function that satisfies *H*(*c*) = *H*(*c*′). This condition is required because the two strands of a molecule have the same coverage by definition. *H*(*c*) represents the coverages of the circular genome associated with the circuit *c*. The *edge multiplicity m*_*c*_(*e*) ≥ 0 of an edge, *e* ∈ *E*, is the number of times the edge is traversed in the circuit *c*, or formally, *m*_*c*_(*e*) = |{*x* ∈ *E*(*c*):*x* = *e*}|. The *edge coverage W*:*E* → ℝ_≥0_ is the weighted sum of the circuit coverages as they traverse edges, or formally, $W(e)=\underset{c\in C}{\sum }m_{c}(e)\cdot H(c)$. The *observed assembly graph* over *H*, denoted by *G*_>0_ = (*V*, *E*_>0_) and with *E*_>0_ = {*e* ∈ *E*:*W*(*e*) > 0}, is *G* restricted to edges with positive coverages. We define *R* = {*c* ∈ *C*:*H*(*c*) > 0}. We refer to the circular genomes associated with circuits for which *H* is positive as the underlying *genome configuration* of the community.

#### Assessing genome coverages using edge coverages

Although genome coverage *H* and its induced genome configuration are latent, edge coverage *W* is an observed variable. We first assume that *W* is known and use it to define dominant cycles. In the implementation section below, we describe how we estimate *W* by mapping paired reads back onto the assembly. The underlying genome configuration of an evolving and complex microbial population may include the repetition of genetic sequences within the same genome or between different genomes. Accordingly, *H* can have a positive value for partially overlapping circuits. Although we allow *H* to have a positive value on any circuit, we focus on characterizing *H* solely over graph cycles. We do this by defining two latent and two observable variables for each cycle as follows.

#### Cycle multiplicity

Let $\bar{x}={\left(x_{i}\right)}_{i=0}^{n_{x}-1}$ be the contigs of a cycle $c=p(\bar{x})$, let $\bar{y}={\left(y_{i}\right)}_{i=0}^{n_{y}-1}$ be the contigs of a circuit $t=p(\bar{y})$, and let *m* ≥ 0 be a nonnegative natural number. We say the *c* has a multiplicity of *m* in *t* if there exists 0 ≤ *s*_*x*_ < *n*_*x*_ and 0 ≤ *s*_*y*_ < *n*_*y*_ such that $x_{\left(s_{x}+i)(mod\;n_{x}\right)}=y_{s_{y}+i}$ for *i* = 0, …, (*m* − 1) · *n*_*x*_. In words, *c* has a multiplicity of *m* in *t* if there exists a subset of *t* that traverses along the edges of *c* while visiting one of the contigs of *c* at least *m* times before leaving the cycle. Note that the final and possibly partial pass along the contigs of *c* is counted toward the multiplicity. By definition, every cycle *c* has a multiplicity of zero in any circuit *t*. We define the *cycle-circuit multiplicity μ*_*t*_(*c*) to be the maximal *m* for which *c* has a multiplicity of *m* in *t*, and define the *cycle multiplicity μ*(*c*) = max_*t*∈*R*_*μ*_*t*_(*c*). In words, *μ*(*c*) is equal to the maximal *cycle-circuit* multiplicity involving *c* for which the circuit *t* satisfies *H*(*t*) > 0.

#### Real and phantom cycles

Cycles are ambiguous in the sense that the precise number of cycle turns involved in producing an associated circular genome (i.e., tandem repeats) cannot be inferred from the graph. To address this inherent ambiguity, we create a proxy for the genome coverage *H* using a generalized genome coverage *ψ* defined as follows: For a cycle *c* = *p*((*x*_0_, *x*_1_, …, *x*_*n*−1_)), we use *c*^*m*^ to denote the circuit produced by *m* ≥ 1 loops along the contigs that define the cycle, or formally, *c*^*m*^ = *p*((*y*_0_, *y*_1_, …, *y*_*m*·*n*−1_)) with *y*_*i*_ = *x*_*i mod n*_ for *i* = 0, …, *m* · *n* − 1. We define the *generalized genome coverage* of a cycle *c* to be $\psi (c)=\underset{m\in N}{\sum }H(c^{m})$ and call a cycle *c* for which *ψ*(*c*) > 0 a *real cycle*; otherwise, we call it a *phantom cycle*.

#### Dominant cycles

Our goal is to distinguish between real and phantom cycles in the face of an unknown and potentially complex background of genomes. This is a nontrivial task, as the assembly graphs of community-based genome configurations may be riddled with phantom cycles. For example, the mere presence of *n* instances of an integrated repeat element results in *n* phantom cycles. We address this by defining dominant graph cycles as follows. Let *c* be a cycle in the graph. Given an edge *e* = (*v*, *u*) we say *e* is an *outgoing edge* of *c* if *v* ∈ *V*(*c*) and e∉E(c), and denote by *E*_out_(*c*) the set of outgoing edges. Similarly, we say *e* is an *ingoing edge* of *c* if *u* ∈ *V*(*c*) and e∉E(c), and denote by *E*_in_(*c*) the set of ingoing edges. We define the *bottleneck coverage σ*(*c*) = min _*e*∈*E*(*c*)_*W*(*e*), the *outgoing coverage* $\tau_{out}(c)=\underset{e\in E_{\mathrm{out}}(c)}{\sum }W(e)$, and the *ingoing coverage* $\tau_{in}(c)=\underset{e\in E_{\mathrm{in}}(c)}{\sum }W(e)$. We define the *external coverage τ*(*c*) = (*τ*_*out*_(*c*) + *τ*_*in*_(*c*))/2 and call the cycle *c* a *dominant cycle* if *σ*(*c*) > *τ*(*c*). We also define the *cycle score* $s(c)=\frac{\sigma (c)}{\tau (c)}\;$.

#### Lower bound on generalized genome coverage

The cycle multiplicity, *μ*, and generalized genome coverage, *ψ*, are two latent variables of graph cycles. The following theorem establishes an association between them as a function of the two observable cycle variables *σ* and *τ*.

**Theorem 1**. Let *X* be a nonredundant contig set, let *G* be the assembly graph of *X*, let *H* be a latent genome coverage. For any cycle *c* in *G*, it holds that *σ*(*c*) − *μ*(*c*) · *τ*(*c*) ≤ *ψ*(*c*).

See formal proof in Supplemental Note 1. In a nutshell, the proof is based on the fact that any circuit other than the cycle itself that contributes to the bottleneck coverage must also contribute to the external coverage on its way into and out of the cycle. The cycle multiplicity accounts for the increased contribution to the bottleneck of circuits that makes multiple turns within the cycle before exiting. A simple corollary of this inequality is that if a cycle is a phantom cycle (i.e., *ψ*(*c*) = 0), then *μ*(*c*) ≥ *s*(*c*). In other words, the multiplicity of a phantom cycle must be greater than or equal to its score.

### Data sets

#### Metagenomic and reference data sets

Reference genome sequences for 100 plasmids, 100 phages, and 155 microbial host genomes were downloaded from NCBI (Supplemental Table 1). Shotgun data for the two focal subjects were downloaded from the NCBI Sequence Read Archive (SRA; https://www.ncbi.nlm.nih.gov/sra) database, accession numbers SRR8187104 (gut sample #1) and SRR8186375 (gut sample #2). The 30 metagenomic data sets used in this work were published in studies involving human stool samples from healthy subjects (The Human Microbiome Project Consortium 2012), a marine environment (Biller et al. 2018), and wastewater (Hendriksen et al. 2019). For each of the three environments, we chose the top 10 samples with the highest read count from unique geographic locations or subjects (Supplemental Table 3). The CAMI Low data set was downloaded from http://gigadb.org/dataset/100344, and the reference circular genomes were considered as the elements in the circular_one_repeat subdirectory.

#### Simulated plasmid and phage configurations

A *genome configuration* is composed of a set of circular genomes with associated x-coverage values. To generate a plasmid configuration, a 50-kb circular random sequence (“central allele”) was generated, and n = 8 circular variants were subsequently generated by introducing for each variant a single-genome rearrangement event on the sequence of the central allele. The rearrangement events were selected in equal probability among insertions, inversions, and deletions. An insertion involved introducing a sequence of a length chosen uniformly from the range 100 bp–10 kb and inserted at a random genome coordinate of the central allele. An inversion or deletion involved a random genome segment with a length that followed a uniform distribution. For a prophage configuration, a 10-kb phage circular genome sequence (“central allele”) and a 1-Mb host chromosome circular genome sequence were generated. The sequence of the phage genome was integrated at n = 8 random locations along the chromosome genome. For both plasmid and phage, the central allele was assigned an x-coverage F between five and 100, increasing in steps of five. For a plasmid configuration, the x-coverage of each variant was assigned a fraction of 100 − F weighted by the beta distribution *Beta*(*α* = 2, *β* = 2). For a phage configuration, the x-coverage of the host chromosome was set to $\frac{100-F}{n}$. For each central allele frequency, 30 configuration replicates were used, producing a total of 1200 configurations for the plasmid and the phage scenarios.

#### Reference-based configurations

The simulated chromosomal metagenome configuration was created using 155 reference bacterial chromosome FASTA files selected from diverse taxa as specified in Supplemental Table 1. Reference sequences in the reference FASTA file that either were <200 kb or contained the “plasmid” keyword in the sequence header were removed. Each reference sequence was assigned an x-coverage sampled uniformly in the range of 10× to 75×. Each reference plasmid or phage genome was run as a separate data set with an x-coverage of 50×.

#### Simulating sequence data

For each specific genome configuration (either phage, plasmid or chromosomal), a *read data set* was generated as follows. The total number of reads for a genome was computed such that the mean x-coverage matched the x-coverage specified in the configuration, taking into account the length of each read side, which was set to 150 bp. A single paired read was generated by selecting a random strand and coordinate on a genome, as well as with a read insertion size (i.e., distance between read sides) that followed a normal distribution with a mean of 200 bp and a standard deviation of 15 bp.

### Implementation overview

We first give a short overview of the implementation before diving into the technical details. We designed an algorithm that recovers all dominant cycles in an assembly graph (Algorithm 1). The algorithm reports only dominant cycles (for proof, see Supplemental Note 2) and does not miss dominant cycles (for proof, see Supplemental Note 3). We implemented a tool (DomCycle) that receives a metagenomic assembly and a set of paired reads mapped to the assembly as input and then outputs all dominant cycles in the assembly graph. DomCycle works by applying first a contig-level step that identifies candidate cycles from the assembly graph. In this step, the edge coverage *W* is estimated using mapped read densities within contigs and between contigs. Then, Algorithm 1 is applied to find a set of candidate cycles. In a second nucleotide-level step, candidate cycles are scrutinized at single-nucleotide resolution. The nucleotide-level step is designed to address issues that arise from assembly limitations outside the scope of the theoretical framework, such as overassembly (concatenated contigs that are nonadjacent in some underlying genomes) and fragmentation (i.e., missing or very short contigs). In this step, candidate cycles are tested using two nucleotide-level criteria that incorporate mapped read densities along the cycles. Broadly speaking, the nucleotide-level definitions of dominant cycles adapt the theoretical definitions to the peculiarities of metagenome assemblies. Based on the previously defined cycle score *s*(*c*), we define a nucleotide-level version called the *global nucleotide-level score*. It is computed by considering all reads that map on both sides to the assembly and on at least one side anywhere along the cycle. A second score, called the *local nucleotide-level score,* was designed to directly address the distribution of singleton reads for which one side did not map to the assembly owing to assembly fragmentation. The local score is designed to filter out cases in which a cycle is not real but was integrated into a larger genomic context through sequences that failed to assemble. To account for stochasticity in read distribution, *P*-values are assigned to the two nucleotide-level scores by modeling the number of reads using a binomial distribution. Candidate cycles for which both global and local nucleotide-level scores are significantly greater than one (*P* < 0.01) are reported as dominant cycles, alongside their corresponding genomes.

Algorithm 1: **Find dominant cycles in graph**

Input: Graph *G*_>0_ = *G*(*E*, *V*, *W*)

Output: Set of dominant cycles R

 1 R=∅;

 2 For each edge *e*_0_ = (*v*_0_, *u*_0_) in *E* do:

 3  *c* = (*e*_0_), v = *v*_0_, u = *u*_0_, *b* = *W*(*e*_0_), a = 0, is_open=true;

 4  while ***a*** < ***b*** and is_open do:

 5   *N* = {*e* = (*x*, *y*) ∈ *E*:*x* = u};

 6   *M* = {*e* ∈ *N*:*W*(*e*) ≥ *b*};

 7   if |***M***| ≠ 1 then break; {*m*} = *M*;

 8   u=tail_vertex(m);

 9   is_open=u∉cycle_vertices(c);

10   push_back(c,m);

11   $a=a+\underset{e\in N\setminus m}{\sum }W(e)$;

12  if ***v*** = ***u*** and ***a*** < ***b*** then insert (*R*,*c*);

13 return *R*;

### Implementation details

#### Basic read processing

All raw reads of a given data set were processed as follows. Exact duplicate reads were removed using a custom in-house C++ program. Sequencing adapter removal and sequencing quality filtering was performed using Trimmomatic (Bolger et al. 2014) (v0.38) with the command parameters “LEADING:20 TRAILING:3 MAXINFO:60:0.1 -phred33.” Reads mapping to the human genome were discarded from downstream analysis using DeconSeq (v0.4.3, hg38 as reference) (Schmieder and Edwards 2011).

#### Genome assembly

Paired-end reads were assembled using MEGAHIT (v1.1.3) (Li et al. 2015) with the parameters “‐‐merge-level 1000,0.95 ‐‐k-min 27 ‐‐k-max 77.” If MEGAHIT did not assemble up to k = 77, the assembly with the greatest *k*-mer size was used. Contigs <231 bp were discarded to ensure external edges are properly assigned weights (see below). To map reads back onto the assembly, read sides were trimmed to include only the first 50 bp of each read side, and sides were mapped separately to the assembly using BWA (v0.7.12) (Li and Durbin 2009). Downstream analysis was limited to *mapped read sides* by filtering out sides for which (1) the match length did not span the entire 50 bp or (2) the mapping edit distance was greater than one. A FASTG was created using the contig2fastg program from the megahit toolkit (v1.1.3).

#### Internal edge weight

Intra-contig reads were classified as *forward-facing*, *back-facing*, *left-facing*, and *right-facing reads* based on the relationship between strands and coordinate order. For example, a read was classified as a forward-facing read if the read side mapping to the *b* = *W*(*e*_0_)*molecule length* (IML) of forward-facing reads was defined as the distance between the two start coordinates of the read sides. M was defined to be the median of the IML distribution. The *max read distance* (MD) was calculated by taking the 99.5th percentile of the distribution of IMLs of 100,000 randomly selected forward-facing reads and adding a safety margin of 200 bp. The x-coverage of a contig was estimated by $\frac{RS\;\times \;RL}{CL}$, where RS is the number of mapped read sides that mapped to the contig, and CL is the contig length. RL was calculated as the average pretrimmed read side length for 1000 randomly selected reads. The two internal edges associated with a contig and its reverse complement were assigned a weight equal to the estimated x-coverage of the contig.

#### External edge weight

External edge weights were calculated based on *external reads*, defined as the union of inter-contig reads and back-facing intra-contig reads. We defined for each external read the *external inferred molecule length* EML = *d*_1_ + *d*_2_ − *k*, where *d*_*i*_ is the distance between the start coordinate of a read side and the coordinate of the contig end that is reached if moving in the strand direction, and k is the *k*-mer length used for assembly (here, k = 77). Each external read was associated with two corresponding external edges, that is, *e*(*x*, *y*) and *e*(*y*′, *x*′). An external read was classified as supporting an external edge (for both associated edges) if (1) EML <MD for the read, and (2) the mapped contig segments were not contained in the segment of (k + 3) bases at the beginning or the end of the associated contig. The weight of an external edge was defined as $\frac{2\;\times \;UPR\;\times \;RL}{M}\times \frac{M+k}{M}$, where *UPR* is the number of reads supporting the edge, and *RL* is the pretrimmed read side length. The last term in the formula is included to account for discarded reads that mapped to the first or last (k + 3) bases of a contig. The weighted assembly graph was composed of vertices and internal edges for all contigs and all external edges that had a positive weight.

#### Classifying reads that map to a candidate dominant cycle

Candidate dominant cycles (*candidates*) were generated from the weighted assembly graph using an implementation of Algorithm 1. In the implementation, the dominance test (condition *a* < *b* on lines 4 and 12) was omitted while generating the candidate cycles and replaced by two nucleotide-level dominance tests, as described below. For a given candidate, all reads for which at least one side mapped to the cycle were classified into one of the following groups. A read was classified as a *cycle-support read* if (1) the two read sides mapped to opposite cycle strands after the relative orientations of the cycle contigs were accounted for and (2) either the read was an intra-contig read with IML ≤ MD or the read bridged a pair of consecutive contig ends along the cycle and EML ≤ MD. If both read sides mapped to contigs in the cycle but conditions 1 or 2 were not satisfied, the read was classified as an *intra-nonsupport read*. If one read side mapped to a contig in the cycle and the other mapped to a contig not in the cycle, then the read was classified as an *inter-read*. The remaining case, in which one read side did not pass our mapping criteria and the other side mapped to the cycle, was classified as a *singleton read.* Read sides classified as intra-nonsupport, inter, or singleton were subclassified into intra-nonsupport-in, intra-nonsupport-out, inter-in, inter-out, singleton-in, and singleton-out based on the relative orientation of the read side that mapped to the cycle and the cycle itself. Together, for a given cycle, there was one class that supported the cycle (“cycle-support”) with a matching read count of *N*_support_ and six nonsupporting read classes with matching read counts: $N_{\mathrm{intra}}^{\mathrm{in}},N_{\mathrm{intra}}^{\mathrm{out}},N_{\mathrm{inter}}^{\mathrm{in}},N_{\mathrm{inter}}^{\mathrm{out}},N_{\mathrm{singleton}}^{\mathrm{in}},N_{\mathrm{singleton}}^{\mathrm{out}}$. The *total nonsupport coverage* (*T*_*c*_) of a candidate *c* was set to $(N_{\mathrm{intra}}^{\mathrm{in}}+N_{\mathrm{intra}}^{\mathrm{out}}+N_{\mathrm{inter}}^{\mathrm{in}}+N_{\mathrm{inter}}^{\mathrm{out}})/2$.

#### Base pair level coverages

For a cycle *p*((*x*_0_, …, *x*_*n*−1_)), any coordinate *t* within a contig *x*_*i*_ was converted to the cycle coordinate $y(t,i)=t+\overset{i-1}{\underset{\;j=0}{\sum }}(L_{j}-k)$, where *L*_*i*_ is the length of *x*_*i*_ and *k* is the *k*-mer size used for assembly. For every read side that mapped to a contig within a cycle, a cycle strand was determined based on the mapped contig strand and the orientation of the contig within the cycle. We define the cycle nucleotides that are *covered* by a read as follows. If the read was classified as a cycle-supporting read, it covered all bases between the two cycle coordinates defined by the start of each read side while taking into account the two facing cycle strands. In the remaining cases, in which a read was classified as an intra-nonsupport, inter, or singleton, read sides contributed separately to their respective coverage profiles. In those cases, a read side covered all cycle coordinates within the segment that started on the cycle coordinate at the start of the read side and ended *M* base pairs away while moving along the appropriate cycle strand. All reads were traversed and the nucleotide-level coverage profile was computed for the seven types: supporting, in-intra, out-intra, in-inter, out-inter, in-singleton, and out-singleton. Each profile was a vector of the form $n_{c}^{P}[i]$, where *c* is a cycle, *P* is the profile type, and *i* is the cycle coordinate.

#### Cycle scores and P-values

For each candidate *c*, we computed the base bottleneck coverage $B_{c}=\mathrm{mi}n_{0\leq i<L(c)}(n_{c}^{support}[i])$, where *L*(*c*) is the length of the cycle *c*. The *global nucleotide-level score* was set to $\frac{B_{c}}{T_{c}}$. To assign a *P*-value for the score, we use a null hypothesis that assumes *T*_*c*_ is distributed according to the binomial distribution $B\left(n=N,\;\;p=\;\frac{B_{c}}{N}\right)$, where *N* is the total number of reads that mapped to the assembly, and compute the probability of sampling a value *x* ≤ *T*_*c*_. The local nucleotide-level score was computed as follows: For each base position *i* in a candidate *c*, we calculated $T_{c}^{local}[i]=\max(n_{c}^{in}[i],n_{c}^{out}[i])$, where $n_{c}^{in}[i]=n_{c}^{in-intra}[i]+n_{c}^{in-inter}[i]+n_{c}^{in-singleton}[i]$ and $n_{c}^{out}[i]=n_{c}^{out-intra}[i]+n_{c}^{out-inter}[i]+n_{c}^{out-singleton}[i]$. The nucleotide-level *local score* was defined to be $\mathrm{mi}n_{0\leq i<L(c)}\left(\frac{B_{c}^{local}[i]}{T_{c}^{local}[i]}\right)$, where $B_{c}^{local}[i]=n_{c}^{support}[i]$. We computed the *P*-value of the null hypothesis $B_{c}^{local}[i_{\min}]\leq T_{c}^{local}[i_{\min}]$ for the coordinate *i*_min_, where the minimal score was achieved using a binomial distribution as for the global score. A candidate was reported as a vetted dominant cycle if the *P*-value was under 0.01 for both the global and local nucleotide-level scores.

### Performance evaluation

#### Effect of thresholds

We evaluated the precision and recall of DomCycle as a function of score thresholds and, separately, minimum contig length thresholds. Minimum contig length thresholds were selected from {1*x*, …, 12*x*}, where *x* is the standard *k*-mer size used for assembly (k = 77). Score thresholds applied to both the global and local scores and were selected from {0.25, 0.5, 0.75, 1, 1.5, 2, 5, 10}. Each reference genome was run as a separate data set when running with an alternative parameter threshold.

#### Effect of assembler

The focal subject sample was assembled with metaSPAdes (part of SPAdes v3.14.1; k = 77) and input to DomCycle. We aligned all MGEs reported by DomCycle using the metaSPAdes or Megahit assemblies as input. Two elements (*e*_*i*_, *e*_*j*_) were clustered into cluster *c*_*k*_ following the clustering procedure for MGEs (MGE Clusters). The reported element overlap was the number of clusters containing a MGE reported using both the metaSPAdes and Megahit assemblies as input.

#### Tool comparison

DomCycle was compared to metaplasmidSPAdes (part of SPAdes v3.14.1) (Antipov et al. 2019), Recycler (v0.62) (Rozov et al. 2017), and SCAPP (downloaded June 2020) (Pellow et al. 2021) on reference-based plasmids, the reference-based phage, and the simulated chromosomal metagenome. Tools were run with default parameters. Recycler and SCAPP were supplied FASTGs generated from the same assemblies used as input for DomCycle, while including contigs <231 bp that are discarded by DomCycle (short contigs are needed by those tools to establish contig–contig adjacencies as they do not leverage paired reads). MetaplasmidSPAdes was run with a maximum *k*-mer size of 77 and with the parameter “‐‐assembly-only” when running a simulated data set. For Recycler and SCAPP, BAM files were generated with BWA and filtered using the view command in SAMtools (v1.9) (Li et al. 2009) with parameters “-bF 0 × 0800” as recommended at https://github.com/Shamir-Lab/Recycler (Nov. 2020). For each tool, we considered output FASTA files for element reporting. For SCAPP, we only considered output sequences classified as “confident” predictions. For metaplasmidSPAdes, we considered the output file “contigs.fasta” for element reporting. For the CAMI Low data set, elements reported by the tools were aligned to the n = 20 reference circular genomes using NUCmer. A reported element corresponded to a reference circular genome if there was >90% ANI (MGE Clusters) and preserved genome sequence order. Additionally, each tool reported a single element from the CAMI Low data set associated with the PhiX genome, which was removed from analysis. For each subset of tools *S*, the report overlap was the number of reference circular genomes aligned to an element reported by each tool in *S*. Furthermore, elements reported by DomCycle, metaplasmidSPAdes, and SCAPP on the focal subject (see Fig. 4C) were aligned to each other in pairs and clustered following the clustering procedure for MGEs (MGE Clusters).

#### Computing scores for metaplasmidSPAdes

Global and local scores were computed for each element reported by metaplasmidSPAdes on the focal subject using an alternative procedure. An alternative procedure was used because a description of the contigs comprising reported elements was not identified in the output files supplied by metaplasmidSPAdes. For both alternative global and local scores, the input reads were aligned to the set of reported elements. The global score was computed by classifying nonsupport reads as the sum of singleton, intra-nonsupport, and inter reads mapping to the cycle, averaged across the two strands. The local score was computed according to the standard procedure, but we note that the missing side of a singleton read may have aligned to a genomic region not contained in the set of elements reported by metaplasmidSPAdes. Accordingly, we term the global score as “harsh” and the local score as “loose.”

#### Performance of runs

Configurations were evaluated in the context of runs, defined as the output of running a DNA data set associated with a configuration using a specific tool. For a given genome configuration, the sequences (FASTA format) of output cycles were aligned to the original genome configuration using NUCmer (run with “‐‐maxmatch”), and NUCmer results were parsed using show-coords (run with “-L 200 -I 99.5”). For each genome in a configuration, we calculated the percentage of the genome covered by each reported cycle in a run. For each reported cycle in a run, we identified the *nearest genome*, defined as the genome with the maximum number of bases covered by the reported cycle. We determined that the genome sequence order of the nearest genome was preserved by a reported cycle if the following two conditions were satisfied: (1) the alignments between the reported cycle and genome occur only on one reported cycle strand and one genome strand and (2) when traversing from the beginning of the genome to the end, every alignment to the reported cycle strictly occurs in the order of the reported cycle sequence.

#### Recall and precision for reference data sets

For reference-based runs shown in Figure 2, a run was classified as successful if one of the reported cycles had an alignment coverage >90% of the length of the reference genome, with preserved genome sequence order. Reference genome sequences of plasmids and phage were grouped into a plasmid and a phage data set collection. The recall of a collection was defined as the number of successful runs divided by the number of genomes in the collection. The precision was defined as the number of successful runs in the collection divided by the total number of reported cycles in the collection.

#### Recall and precision for simulated data sets

For the simulated plasmid and phage configurations shown in Figure 3, a run was classified as successful if one of the reported cycles had an alignment coverage on the central allele >98%, with preserved sequence order. The recall value associated with a configuration associated with a specific central allele frequency was set to the number of successful runs divided by the number of replicates (n = 30 replicates were used). Precision was defined as the number of runs aligning to one of the genomes in the configuration with coverage >98% and sequence order preserved divided by the number of reported cycles. If no cycles were reported, precision was defaulted to 100%.

### Cycle characterization

#### Gene annotation

Genes were predicted on dominant cycles using Prodigal (v2.6.3) (Hyatt et al. 2010). Translated gene predictions were aligned to UniRef100 (downloaded July 2020) (Suzek et al. 2007) using DIAMOND (Buchfink et al. 2015) BLASTP with “–sensitive” and a maximum e-value of 0.001. For a gene with multiple alignments to UniRef100, the alignment with the greatest sequence identity was kept, where identity is defined as the alignment similarity multiplied by the fraction of the target gene that was covered by the alignment. HMMER hmmscan (http://hmmer.org v3.1.b2) was used to report cycle gene alignments to the Pfam database (downloaded August 2019) (Finn et al. 2014) with a maximum e-value of 0.001.

#### Cycle classification

The names of gene hits in the UniRef100 and Pfam databases were used to classify dominant cycle into one of the following functional categories: plasmid, phage, mobile, or undefined. A cycle was assigned to a functional category if one or more of its genes matched one of the following regular expressions, tested while accepting both lower and upper case. The *plasmid* category expressions used were “plasmid,” “conjug.*,” “trb$,” “Mob[A-E]$*,” and “Par[A-B].” The *phage* category expressions were “capsid,” “phage.*,” “tail,” “head,” “tape,” “antitermination,” “virus.*,” “bacteriophage,” “sipho*,” “baseplate,” “T4-like.*,” and “myovir.*.” The *mobile* category expressions were “transpos.*,” “resolvase,” “toxin,” “antitoxin,” “excision*,” “integrase,” “relaxase,” “recombination,” “segregation,” “extrachromosomal,” “mobilization,” and “partitioning.” If a cycle met classification for more than one category, then the cycle was assigned to the first matched category in the list: *phage*, *plasmid*, *mobile.* If a cycle matched none of the three categories, it was assigned to the *undefined* category. Circulating cycles (i.e., associated with one of the 20 clusters) were further annotated as described below.

#### Cycle coverages and APs

We define the AMC of a dominant cycle *c* to be *M*_*c*_ − *T*_*c*_ where *M*_*c*_ denotes the median base pair support. For each sample separately, we generated *pseudo-dominant genomes* (PDGs) as follows. We considered *candidate PDGs* as contigs that do not contribute to a dominant cycle and were larger than 2*MD. Profiles were computed for candidate PDGs using the same read classification procedure used for candidates. The PDG base bottleneck coverage was computed while avoiding contig sides, or formally, $B_{p}^{\prime }=\mathrm{mi}n_{MD\leq i<(L(c)-MD)}(n_{p}^{support}[i])$. The *total nonsupport coverage* of PDGs $\left({T^{\prime }}_{p}\right)$ was also computed while avoiding the first and last MD bases on the contig. The *global nucleotide-level score* of PDGs was set to $\frac{{B^{\prime }}_{p}}{{T^{\prime }}_{p}}$. Similarly, the local score for a candidate PDG was computed while omitting positions that were up to MD bases from contig ends. A candidate PDG was classified as a vetted PDG if it passed the global nucleotide score test and the local score test. The AMC of a vetted PDG *p* was set to $M_{p}^{\prime }-T_{p}^{\prime },\;$ where $M_{p}^{\prime }$ is the median support coverage computed over contig bases between the first and last MD bases. The *abundance percentile* of a dominant cycle was set to the percentile of the AMC of the cycle within the distribution of AMC values across all PDGs in the sample. One-sided Kolmogorov–Smirnov tests were used to test whether a distribution of dominant cycle AMCs was enriched over a background of AMCs. To calculate the coverage distribution of contigs in the assembly, we calculated the median coverage for each PDG along the bases between the first and last MD bases in the contig.

### Cycle clustering

#### MGE clusters

The corresponding genomes of all dominant cycles recovered from the 32 samples described in this study were aligned in pairs using NUCmer (run with “‐‐maxmatch” part of the MUMmer v3.1 package) (Kurtz et al. 2004). Single-nucleotide polymorphisms (SNPs) between cycle pairs were identified using show-snps (part of MUMmer). The alignment metrics for two cycles, *c*_*i*_ and *c*_*j*_, were defined as follows. The *alignment fraction* was $F(c_{i},c_{j})=\left(\frac{O(c_{i})}{L(c_{i})}+\frac{O(c_{j})}{L(c_{j})}\right)/\;2$, where *O*(*c*) denotes the number of bases in cycle *c* covered by the alignment, and *L*(*c*) denotes the length of cycle *c*. The *alignment identity I*(*c*_*i*_, *c*_*j*_) was the ANI within the aligned fraction. The *weighted alignment identity* (referred to as the ANI in the main text) was set to *A*(*c*_*i*_, *c*_*j*_) = *F*(*c*_*i*_, *c*_*j*_) × *I*(*c*_*i*_, *c*_*j*_), effectively counting nonaligned regions as having zero identity. The *genome distanc*e used to cluster cycles was *D*(*c*_*i*_, *c*_*j*_) = 1 − *A*(*c*_*i*_, *c*_*j*_). Cycles were clustered based on their genome distances using a threshold of 0.05 (equal to 95% ANI) and using single linkage. A single cluster associated with the PhiX genome and all clusters with a single member were discarded from the analysis. All cycles associated with multimember clusters were termed *circulating cycles*.

#### PLSDB references

A representative cycle for each cluster in a circulating species was aligned to PLSDB (Galata et al. 2019) using “mash dist” (distance cutoff of 0.25) through the PLSDB webserver. Each PLSDB reference was downloaded and aligned using NUCmer, and genome distances were computed in pairs between all reference sequences and the genomes of circulating cycles, as described above.

#### Annotation of circulating cycles

For each cycle *c* belonging to a multimember cluster, we predicted genes by triplicating the cycle sequence and concatenating each copy together sequentially. We only considered genes on cycle *c* where the gene start coordinate *x* (on the triplicated cycle sequence) satisfied *L*(*c*) < *x* ≤ 2 × *L*(*c*). In addition to the annotation procedure described in the section “Gene annotation” above, HMMER hmmscan was used to align (with a max e-value of 0.001) genes to families of mobilization genes, secretion systems, and conjugation genes downloaded from COPLA (https://castillo.dicom.unican.es). Separately, Pfam and UniRef gene classifications were inspected for toxin–antitoxin genes and replication genes. The complete annotations of all genes on circulating cycles are found in Supplemental Table 5. The MOB column in Table 1 was determined based on Relaxase HMM hits to the mobilization, secretion, or conjugation gene families obtained from COPLA. The “uncharacterized” column in Table 1 was determined based on the number of genes that had no HMM/Pfam hits and either had no hits in UniRef or matched an uncharacterized or hypothetical protein. The addiction column in Table 1 was determined based on toxin–antitoxin Pfam and UniRef annotations and included descriptions such as “antitoxin Phd_YefM,” “YoeB-like toxin,” “ParE toxin,” “antitoxin VbhA,” “antidote-toxin recognition MazE,” “PemK-like, MazF-like toxin,” and “RelE toxin.” The replication column in Table 1 was determined based on the Pfam and UniRef descriptions “replication protein,” “RepB family,” “initiator replication protein,” “replication factor-A C terminal domain,” and “firmicute plasmid replication protein (RepL).” The 16 clusters that had a MOB or replication gene were classified as plasmids. Clusters M6, M12, M13, and M15 were left undetermined.

#### Phylogenetic trees

The phylogeny trees in Figure 6 were generated as follows. Given a reference “pivot” cluster member, all SNPs that separated the pivot member and other cluster members (identified by NUCmer) were used to generate a multisequence alignment based on the reference genome of the pivot member. In case of an indel (denoted by a “.” by show-coords of NUCmer), a gap was placed in the alignment. PhyML (Guindon et al. 2010) was run on the alignment with default parameters and optionally marking an outgroup (see below). In the case of M1, the three members of M1b were marked as outgroup members when running PhyML for visualization purposes. Similarly, the *Enterobacter hormaechei* reference was marked as an outgroup for M18.

## Data access

The genomes of the ecMGEs reported in this study have been submitted to the third-party annotation section (TPA) of the NCBI GenBank database (https://www.ncbi.nlm.nih.gov/genbank/) under accession numbers BK061279–BK061297. The source code for DomCycle is available as Supplemental Code and at GitHub (https:// github.com/nshalon/DomCycle).

## Supplementary Material

Supplemental Material
