# Supplementary material for: Precise genotyping of circular mobile elements from metagenomic data uncovers human-associated plasmids with recent common ancestors
Source: Genome Res. 2022 May;32(5):986–1003. doi: 10.1101/gr.275894.121 (PMC9104695; doi:10.1101/gr.275894.121)
Supplement: Supplemental Material [file supp_32_5_986__DC1.html]

Precise genotyping of circular mobile elements from metagenomic data uncovers human-associated plasmids with recent common ancestors — Supplemental Material 

# Precise genotyping of circular mobile elements from metagenomic data uncovers human-associated plasmids with recent common ancestors

## Supplemental Material

- Supplemental\_figs.pdf
- Supplemental\_Material.zip
- Supplemental\_tables.xlsx
- SupplementalNotes.docx
