# Supplementary material for: Precise genotyping of circular mobile elements from metagenomic data uncovers human-associated plasmids with recent common ancestors
Source: Genome Res. 2022 May;32(5):986–1003. doi: 10.1101/gr.275894.121 (PMC9104695; doi:10.1101/gr.275894.121)
Supplement: Supplemental Material [file supp_gr.275894.121_Supplemental_figs.pdf]

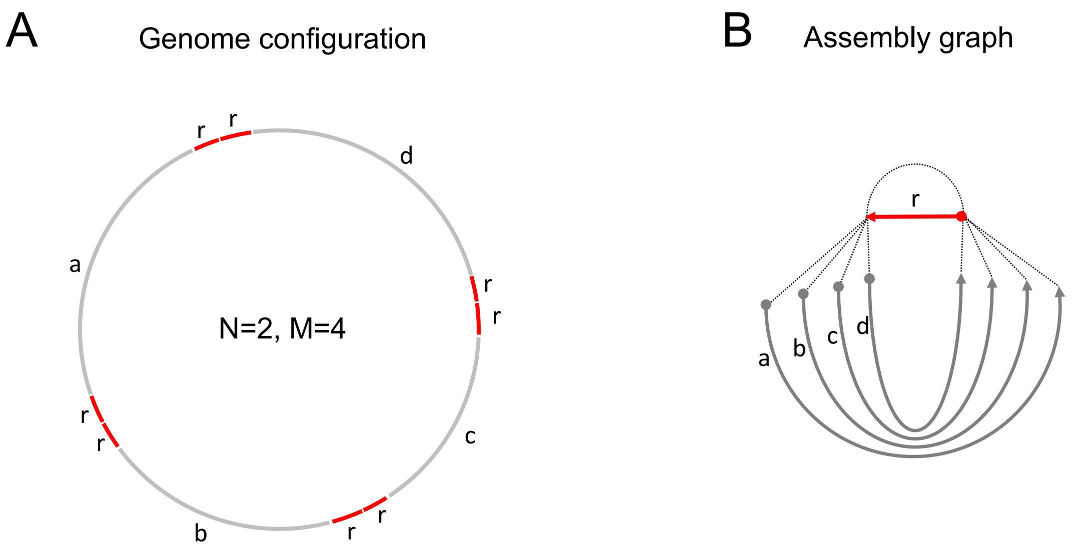


C

| Repeats (N) | Sites (M) | DC_class | DC_score | DC_cycle_count | mpSpades_cycle_count | Recycler_cycle_count |
|---|---|---|---|---|---|---|
| 2 | 1 | not_dominant | 0.828 | 0 | 0 | 1 |
| 2 | 2 | not_dominant | 0.877 | 0 | 1 | 0 |
| 2 | 4 | none_found | NA | 0 | 1 | 0 |
| 2 | 10 | not_dominant | 0.834 | 0 | 1 | 1 |
| 2 | 50 | none_found | NA | 0 | 1 | 0 |
| 3 | 1 | not_dominant | 0.389 | 0 | 2 | 2 |
| 3 | 2 | dominant | 1.532 | 1 | 1 | 2 |
| 3 | 4 | dominant | 1.748 | 1 | 1 | 1 |
| 3 | 10 | dominant | 1.738 | 1 | 1 | 1 |
| 3 | 50 | dominant | 1.817 | 1 | 1 | 1 |

**Supplementary Figure 1. Improved precision of DomCycle when compared to metaplasmidSPAdes and Recycler on tandem repeats.** The 3 tools were tested on 10 negative control datasets. In each dataset, a 10kb sequence r was repeated N consecutive times, and each unit was integrated M times in random location into a genome of length 1Mb. The 4 stretches of DNA between the tandem repeats are denoted a,b,c,d. The x-coverage of the resulting single circular genome was fixed at 50x. **A)** An example of a configuration in which N=2 and M=4. See legend of Figure 1 for graphical details. **B)** The assembly graph for the example in panel A. **C)** The performance of the 3 tools is shown for the 10 datasets. The DC_class column is the result of DomCycle (dominant: dominant cycle found, not_dominant: one or more candidate cycles were found but discarded due to low score, none_found: No candidate cycles found). The DC_score is the maximal score of the candidate cycles. The 3 right columns are the number of cycles reported by the 3 tools (DC: DomCycle, mpSpades: metaplasmidSPAdes). Note that DomCycle successfully avoids reporting all of the 2-tandems and reports some 3-tandems with scores <2. These results partially explain why DomCycle achieves better precision.

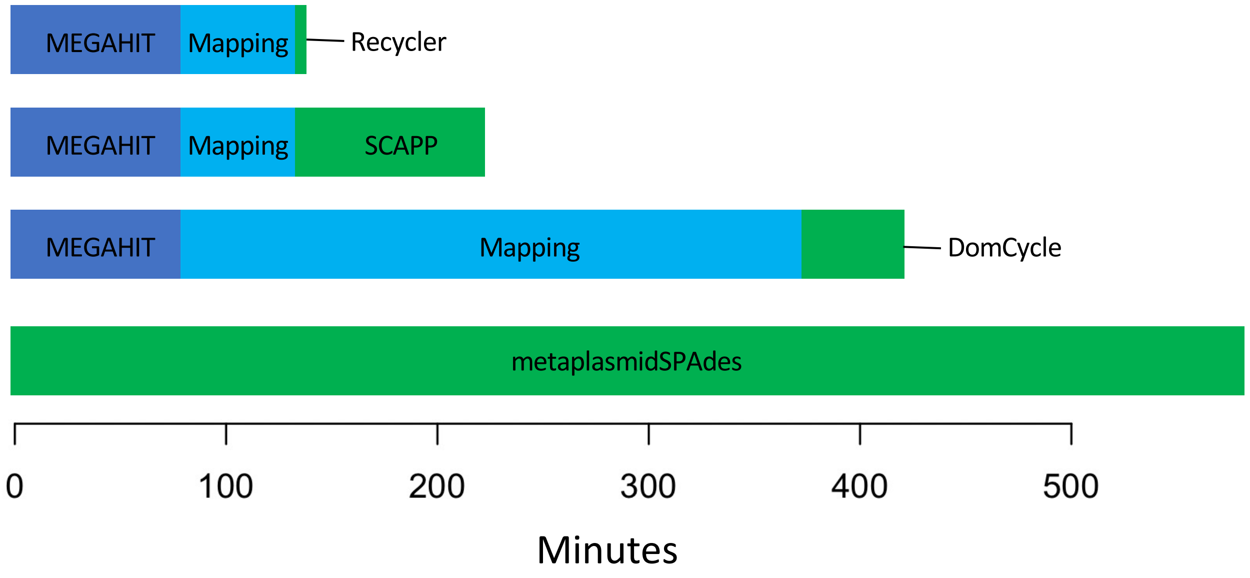


**Supplementary Figure 2.** Runtime for the entire pipeline of DomCycle, SCAPP, Recycler and metaplasmidSPAdes. All tools were on a machine with 1TB RAM and 80 CPU cores. Note that current implementations of DomCycle, SCAPP and Recycler are single threaded. All tools were benchmarked on the simulated dataset derived from 155 bacterial chromosomal genomes described in the paper. Recycler, SCAPP and DomCycle share the assembly step performed using MEGAHIT. Recycler and SCAPP share the read pre-processing step ('mapping'). The read pre-processing step was performed on a single machine and can be accelerated using a distributed approach, which is outside the scope of this work. MetaplasmidSPAdes performs the entire process, from raw reads to genomes of putative mobile elements, in one step.

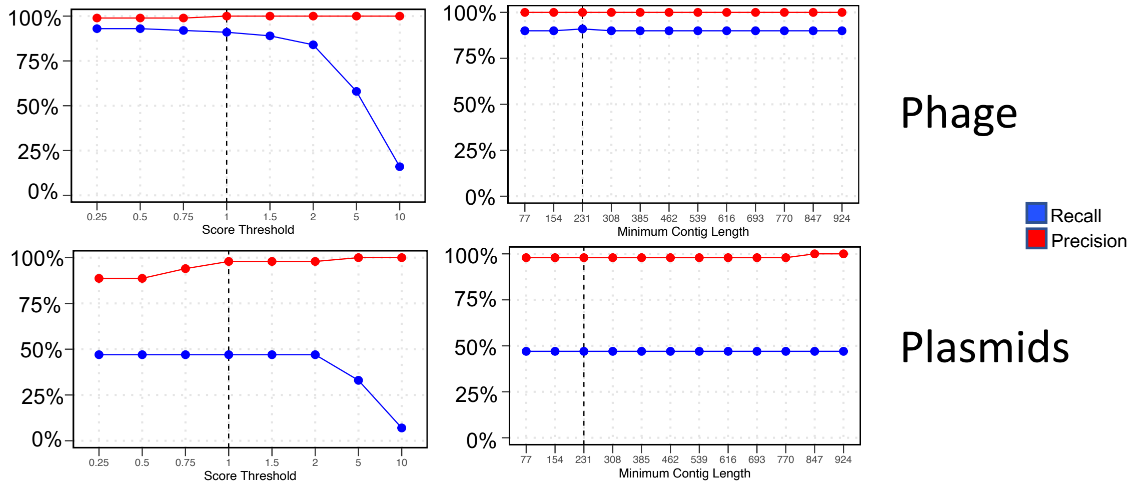


**Supplementary Figure 3.** Recall and precision of DomCycle are robust to the choice of parameter thresholds. Recall and precision for the set of reference phages and plasmids are reported across a parameter scan for score threshold (applied to the local and global scores) and minimum contig length included in the input assembly. Minimum contig lengths are some multiple of the standard assembly k-mer size (k=77).

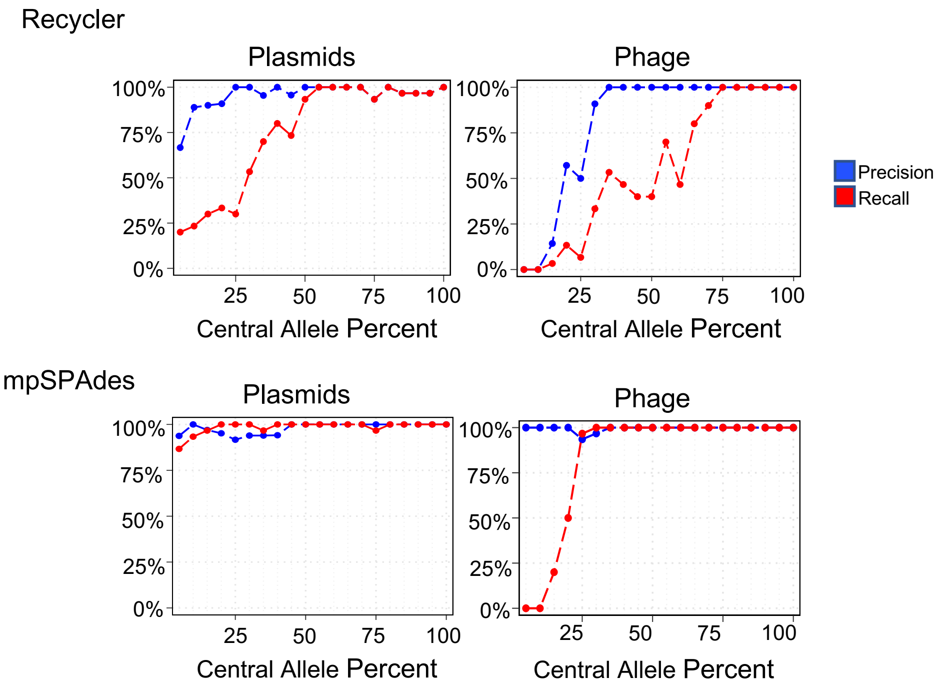


**Supplementary Figure 4.** Performance of Recycler and metaplasmidSPAdes on simulated scenarios. Recall and precision for the set of simulated recombing plasmids (left) and integrating phage (right) for Recycler (top) and metaplasmidSPAdes (bottom).

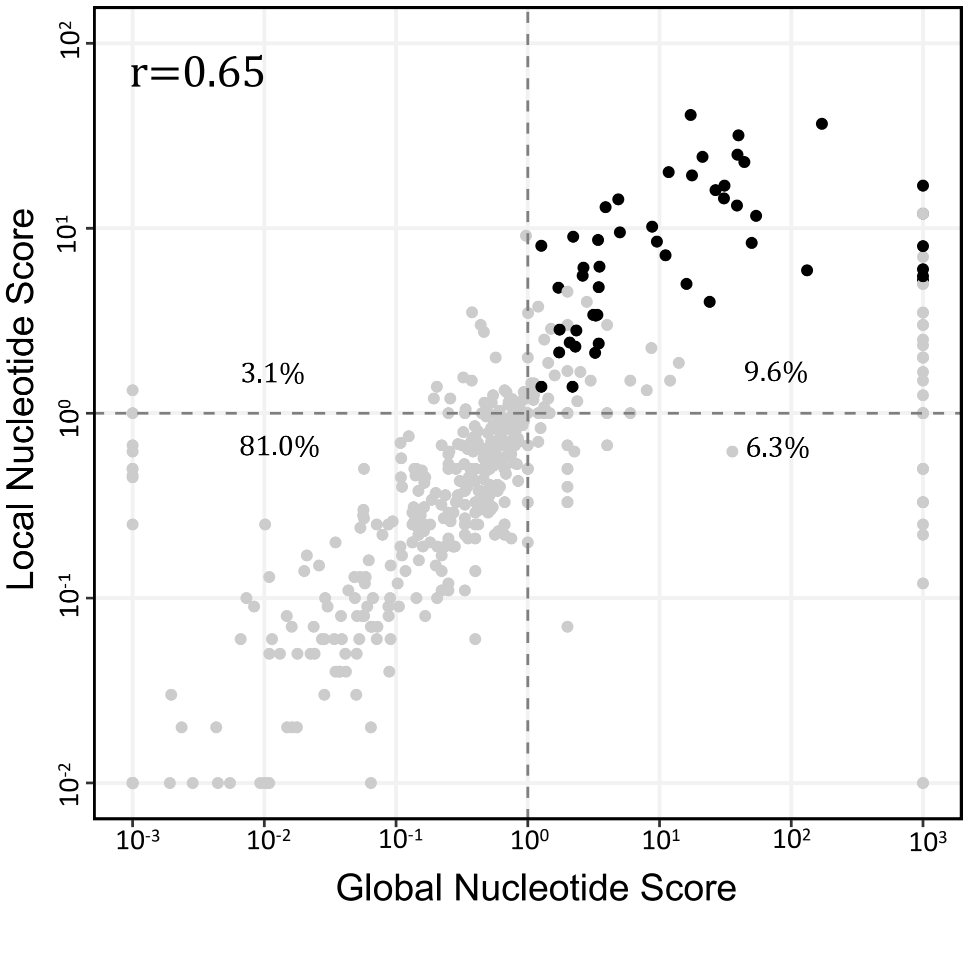


**Supplementary Figure 5.** The distribution of the global nucleotide score and local cycle score for each candidate cycle reported on the gut sample from a healthy adult (SRR8187104). Vetted dominant cycles are shown in black and candidate cycles filtered out by either the global nucleotide score test or the local cycle score test are shown in grey. Horizontal dotted lines drawn show the lower-bound threshold for classifying vetted dominant cycles without accounting for significance through p-values in both score tests. The Pearson correlation coefficient is computed between candidate cycle's global nucleotide score and local cycle score.

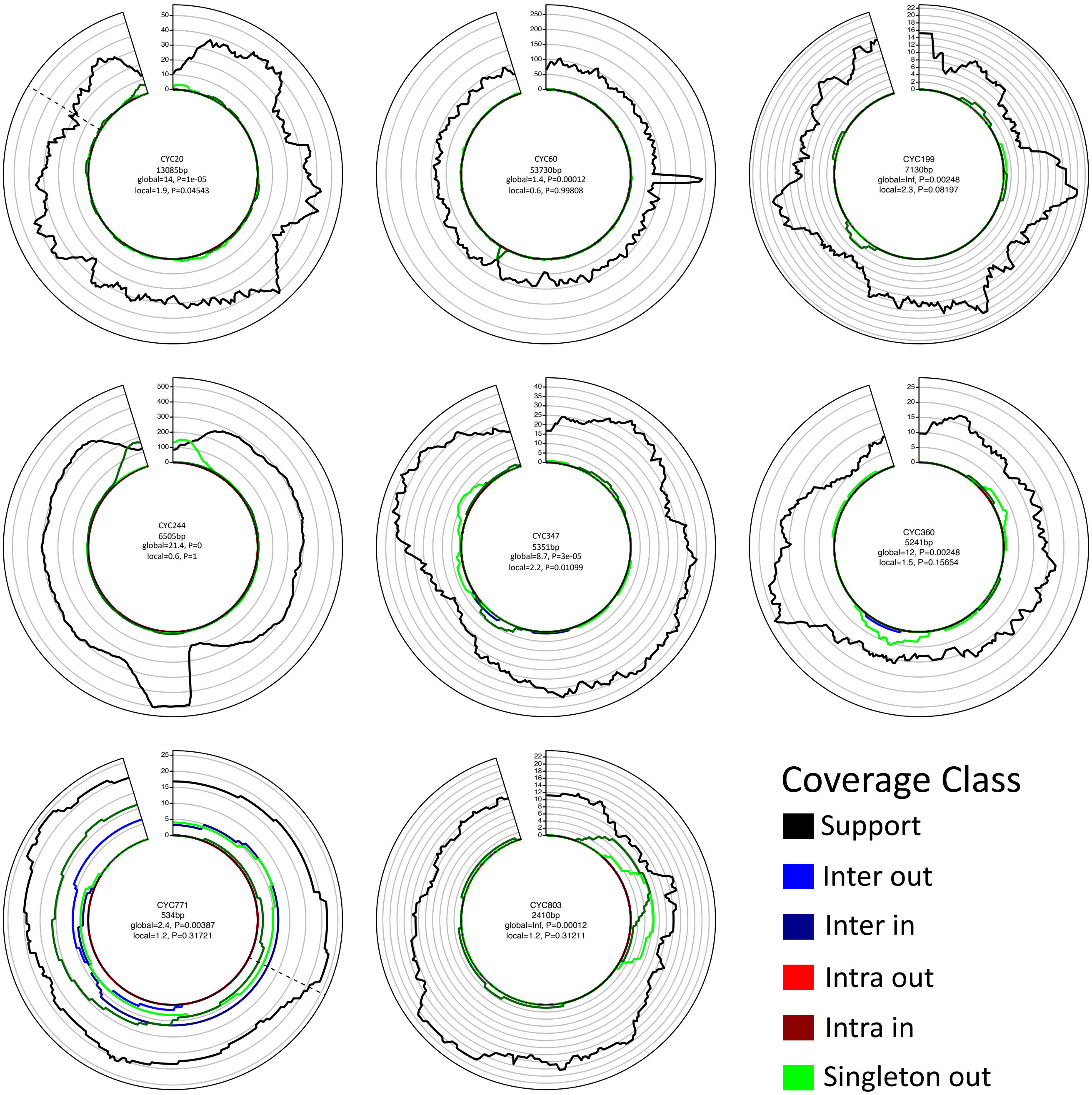


**Supplementary Figure 6.** Examples of candidate cycles filtered out due to poor local nucleotide-level scores in the gut of a healthy adult (SRR8187104). The local nucleotide-level score test calculates the p-value for the hypothesis that the support coverage is greater than the total base pair non-support profile at each base in the candidate cycle (see **Methods**). In comparison to the global nucleotide-level score, the local score accounts for the singleton coverage and tests significance at each candidate cycle base. For instance, CYC244 (middle left) has out singleton coverage (light green) that exceeds the support coverage at the beginning of the cycle; accordingly, this cycle receives a non-significant local nucleotide-level score ($p > 0.01$) at the bases where the singleton out coverage exceeds the support. Intuitively, the high density of singleton reads on CYC244 indicates that there was assembly fragmentation near the contig junction at the beginning (and end) of the cycle. We conservatively assume that the missing singleton read side originated from a sequence that is missing in the assembly. Thus, we cannot confidently conclude that CYC244 is dominant. A similar rationale extends to other candidate cycles depicted.

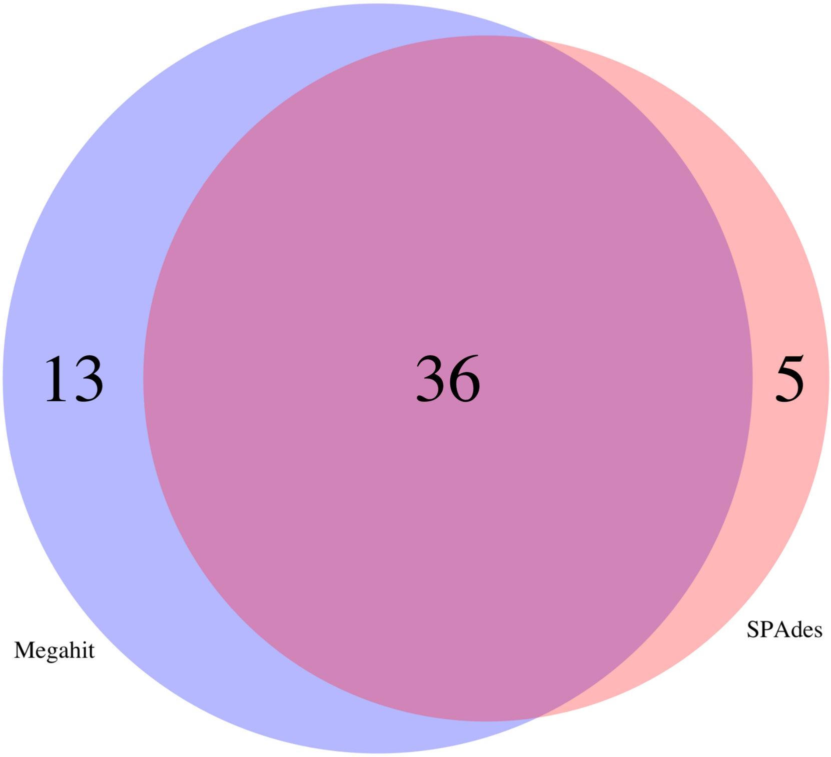


**Supplementary Figure 7.** Genome clustering for cycles reported by DomCycle based on an input MEGAHIT assembly (left) or metaSPAdes (right). The number of shared cycles was calculated by clustering the union of MEGAHIT and metaSPAdes cycles, then computing the number of MEGAHIT-reported cycles which share a cluster with a metaSPAdes-reported cycle.

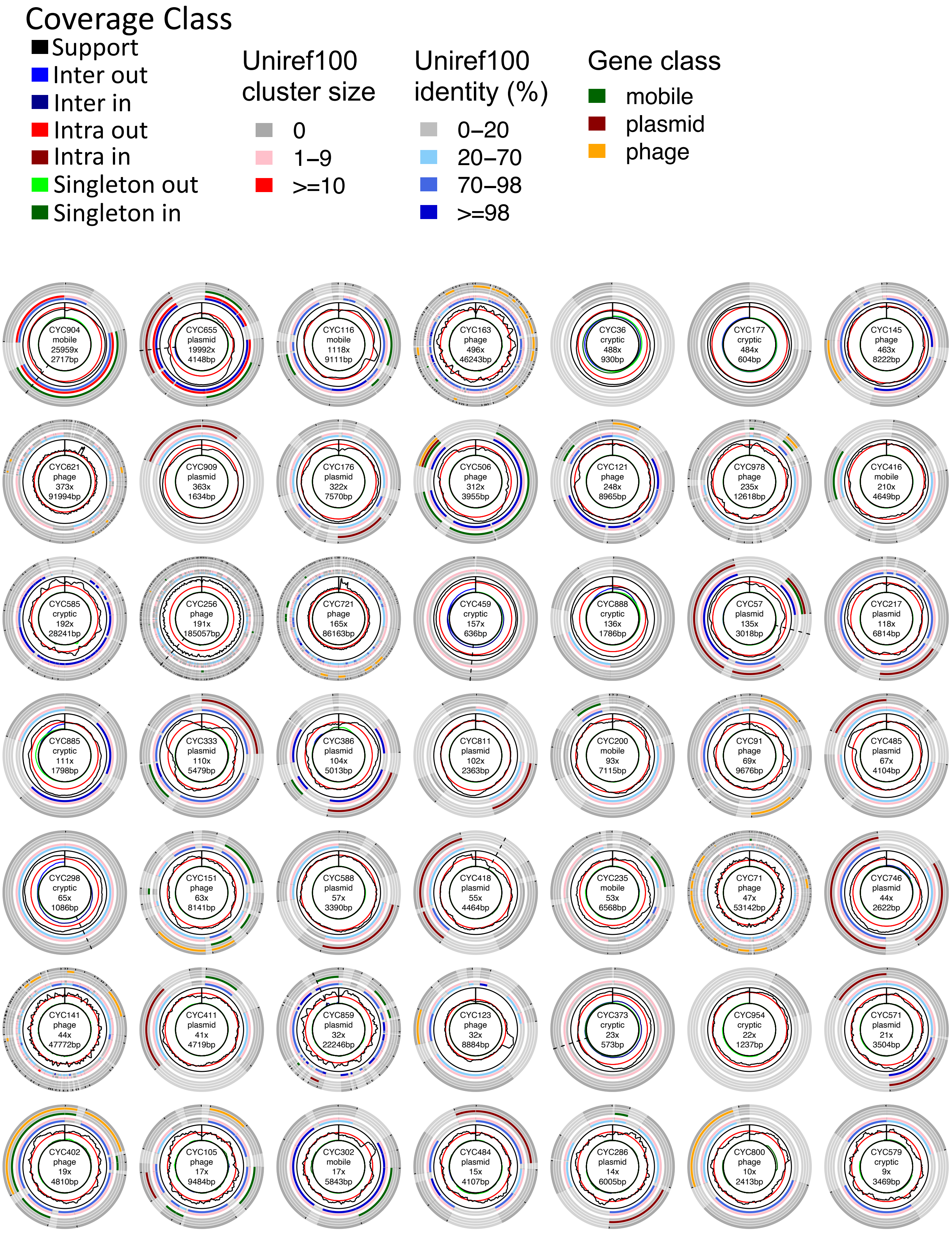


**Supplementary Figure 8.** All ecMGEs identified in the gut of pilot subject 1. Each plot shows the coverage profile, gene positions, gene identity, Uniref cluster size, and gene classification for each cycle.

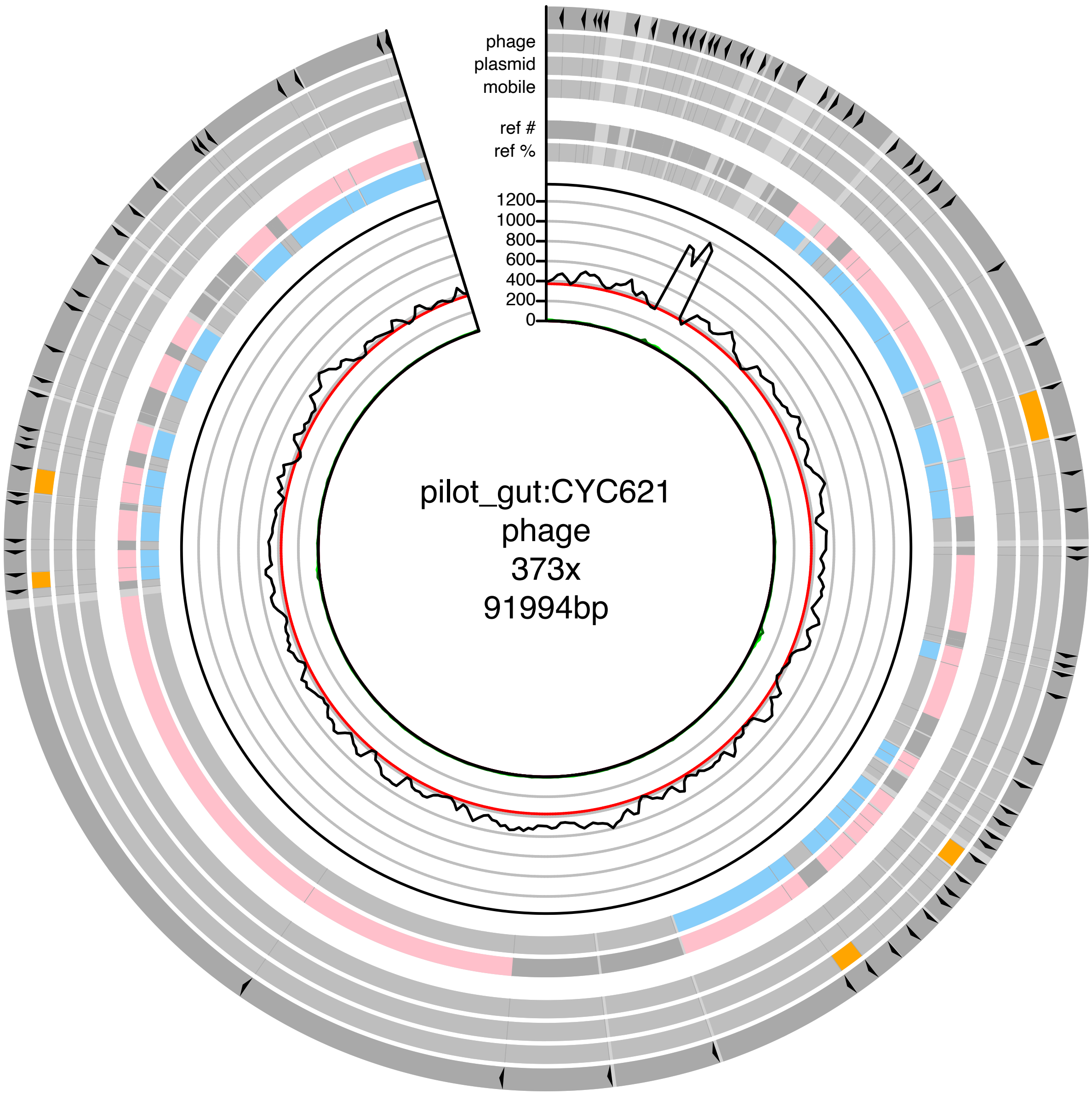


**Supplementary Figure 9.** CrAssphage-like element identified in the gut of the central subject in the study. See Figure 4A for color legend.

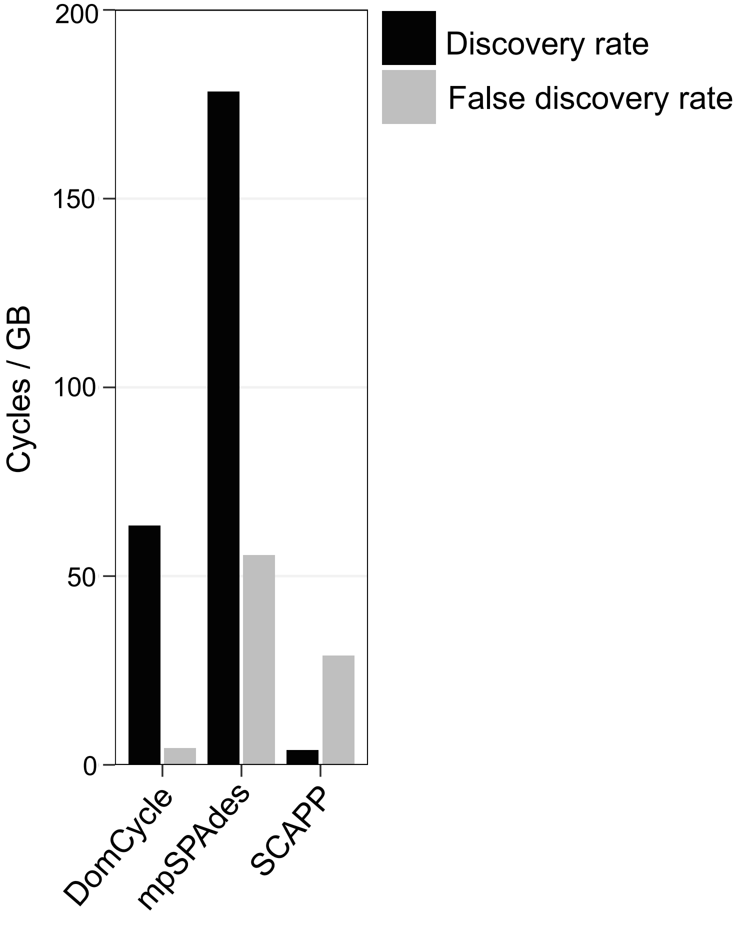


**Supplementary Figure 10.** We used the simulated chromosomal configuration benchmark in Figure 2 to estimate the false-positive rate of DomCycle, metaplasmidSPAdes, and SCAPP. The false-positive rate is significant compared to the element reporting rate on the central subject for metaplasmidSPAdes and SCAPP.

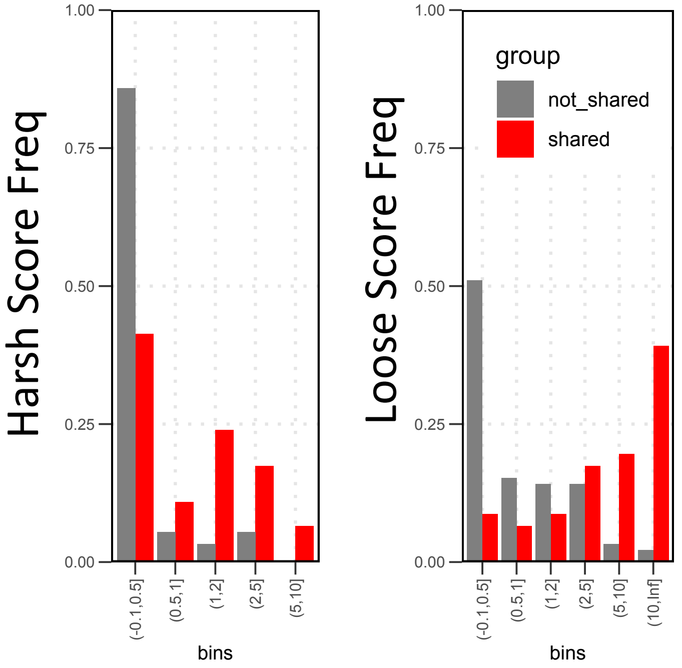


**Supplementary Figure 11.** Harsh and loose scores were calculated on the set of elements reported by metaplasmidSPAdes on the focal subject. These scores were computed based on alternate read mapping and scoring procedure meant to reflect the DomCycle system most thoroughly (**Methods**)**,** but adapted to the supplied metaplasmidSPAdes output. The distribution of harsh scores (left) and loose scores (right) for cycles reported by metaplasmidSPAdes. Cycles reported by metaplasmidSPAdes were aligned to cycles reported by DomCycle and categorized as either reported by DomCycle (red) or not (gray). Cycles reported by metaplasmidSPAdes and DomCycle have higher scores than cycles reported only by metaplasmidSPAdes.

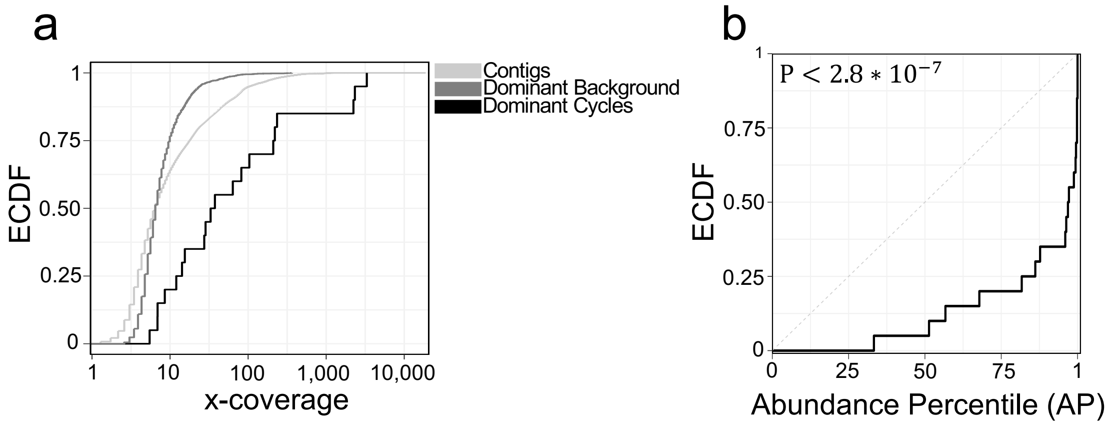


**Supplementary Figure 12.** Results for a second deeply-sequenced gut sample from a healthy human adult (SRR8186375) recapitulate trends shown in Figure 4. **(a)** Empirical cumulative density functions (ECDF) for the median support coverage distribution for candidate pseudodominant genomes (light grey), the adjusted median coverage (AMC) distribution for pseudodominant genomes (dark grey), and AMC distribution for dominant cycles (black). **(b)** ECDF for the abundance percentiles (AP) of dominant cycles. The AP for each cycle is computed to be the percentile of the cycle AMC in the background distribution of AMCs among pseudo-dominant genomes. The dotted line shows the AP ECDF for pseudo-dominant genomes (KS-test, $\mathrm{P} < 2.8 * 10^{-7}$).

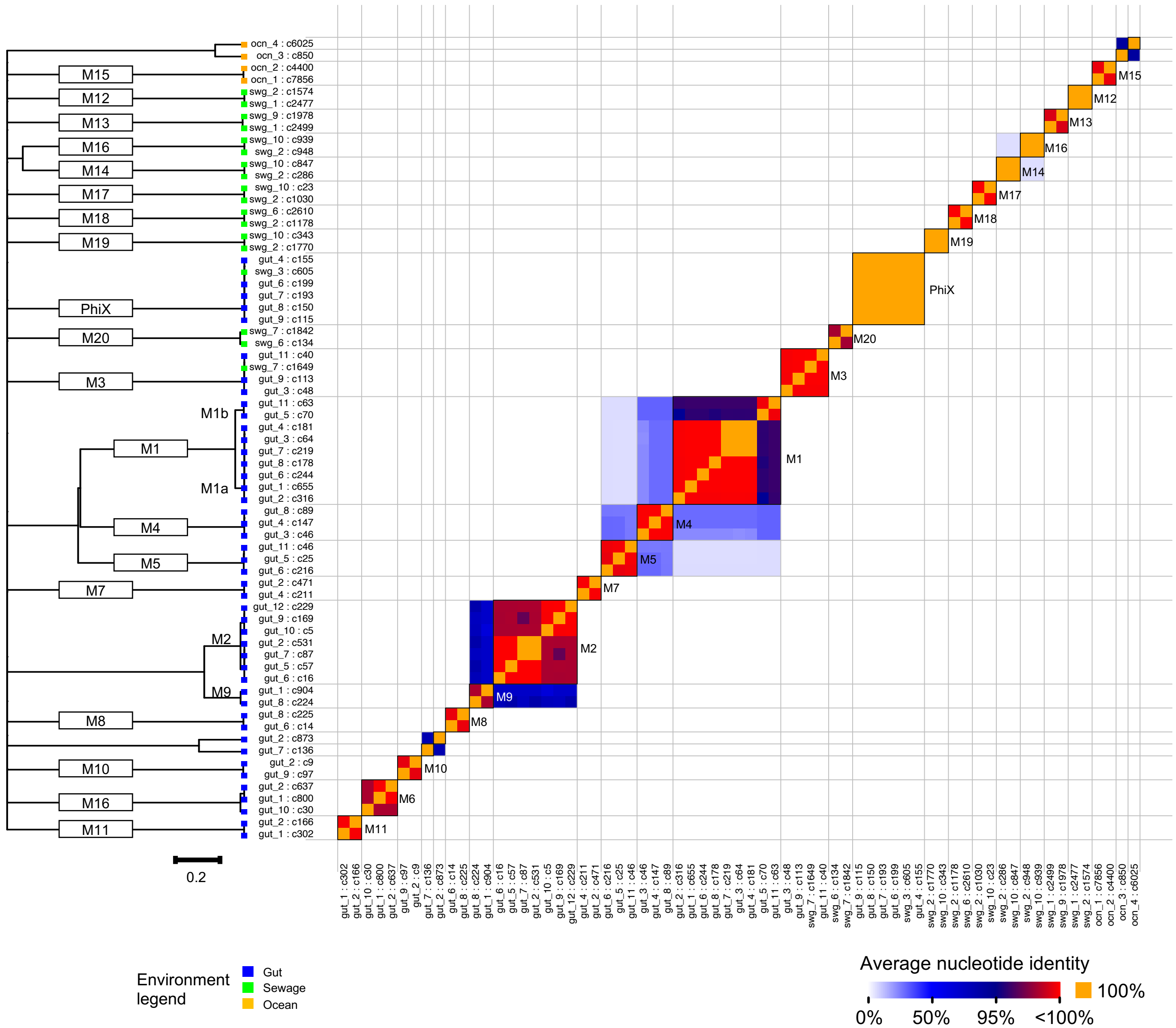


**Supplementary Figure 13.** The 286 eMGEs were clustered using hierarchical clustering performed with single linkage. Shown are eMGEs for which the distance to their nearest neighbor was under 0.5 (i.e., >50% ANI). Left shows clustering dendrogram, with a scale bar showing a distance of 0.2 (equivalent to 80% ANI) and nodes colored by environment. Matrix squares colored by sequence identity, with perfect alignments (100% ANI) highlighted in orange. The 20 multi-member clusters (threshold 95% ANI), numbered M1 to M20, are marked on the plot. The PhiX cluster and all single-member clusters were omitted from downstream analysis.

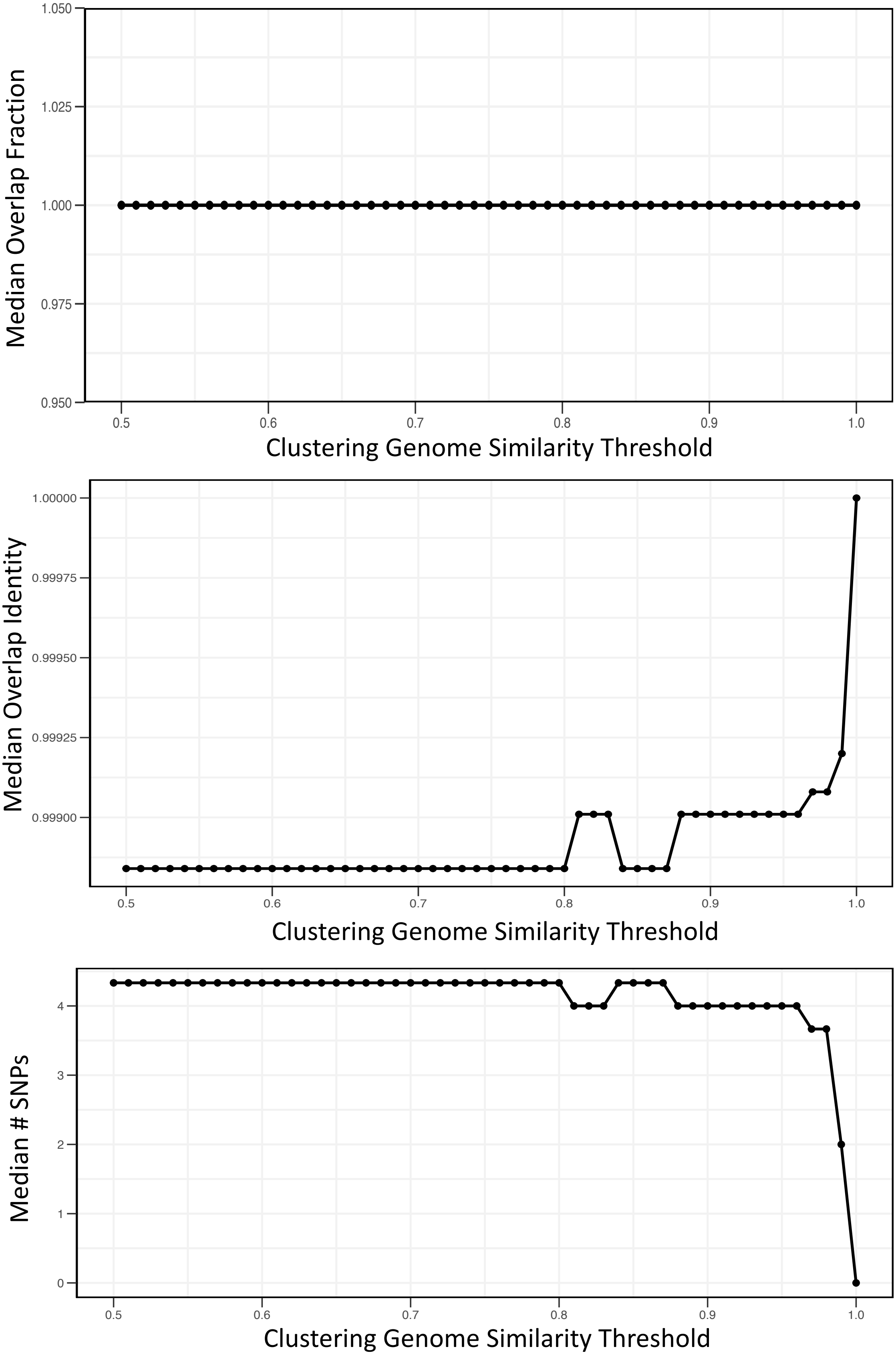


**Supplementary Figure 14.** The median of each intra-cluster mean metric as a function of clustering genomic similarity threshold. The three metrics for cluster tightness are robust to changes in the minimum genome similarity clustering threshold.

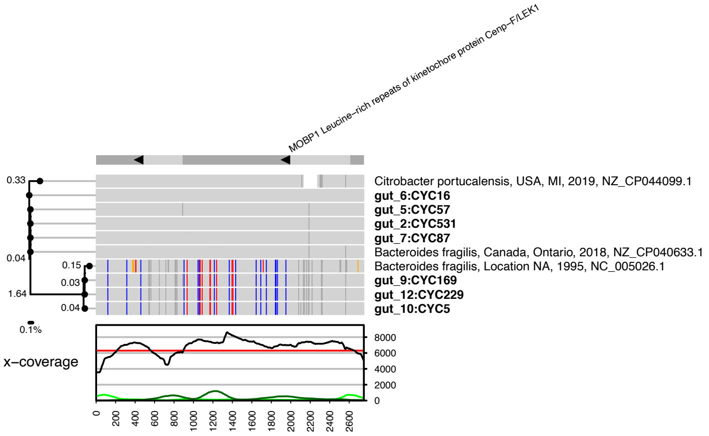


M2, gut_6:CYC16

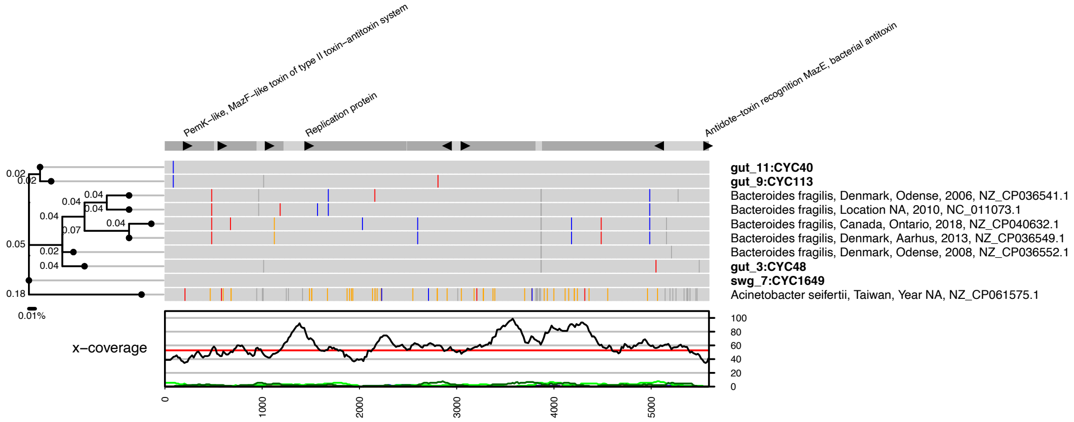


M3, swg_7:CYC1649

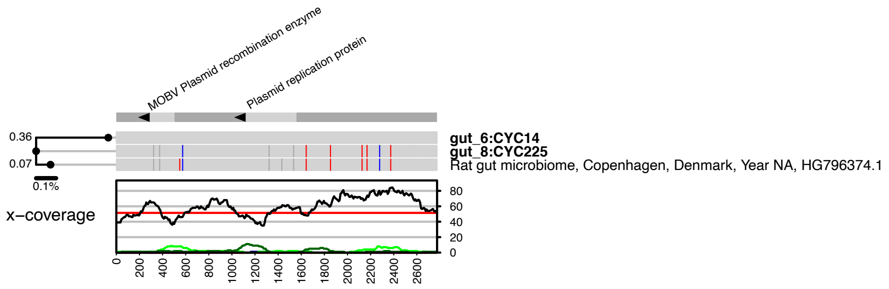


M8, gut_6:CYC14

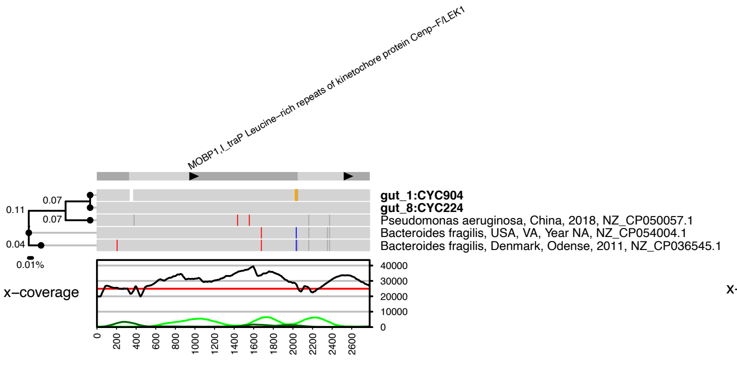


M9, gut_8:CYC224

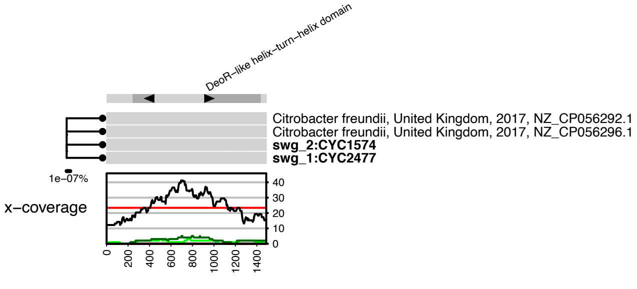


M12, swg_1:CYC2477

**Supplementary Figure 15.** Clusters with one or more references in PLSDB. Legends as in Figure 6. The 5 clusters were found in multiple environments (human gut, sewage and rat microbiome) and are associated with diverse microbial hosts.

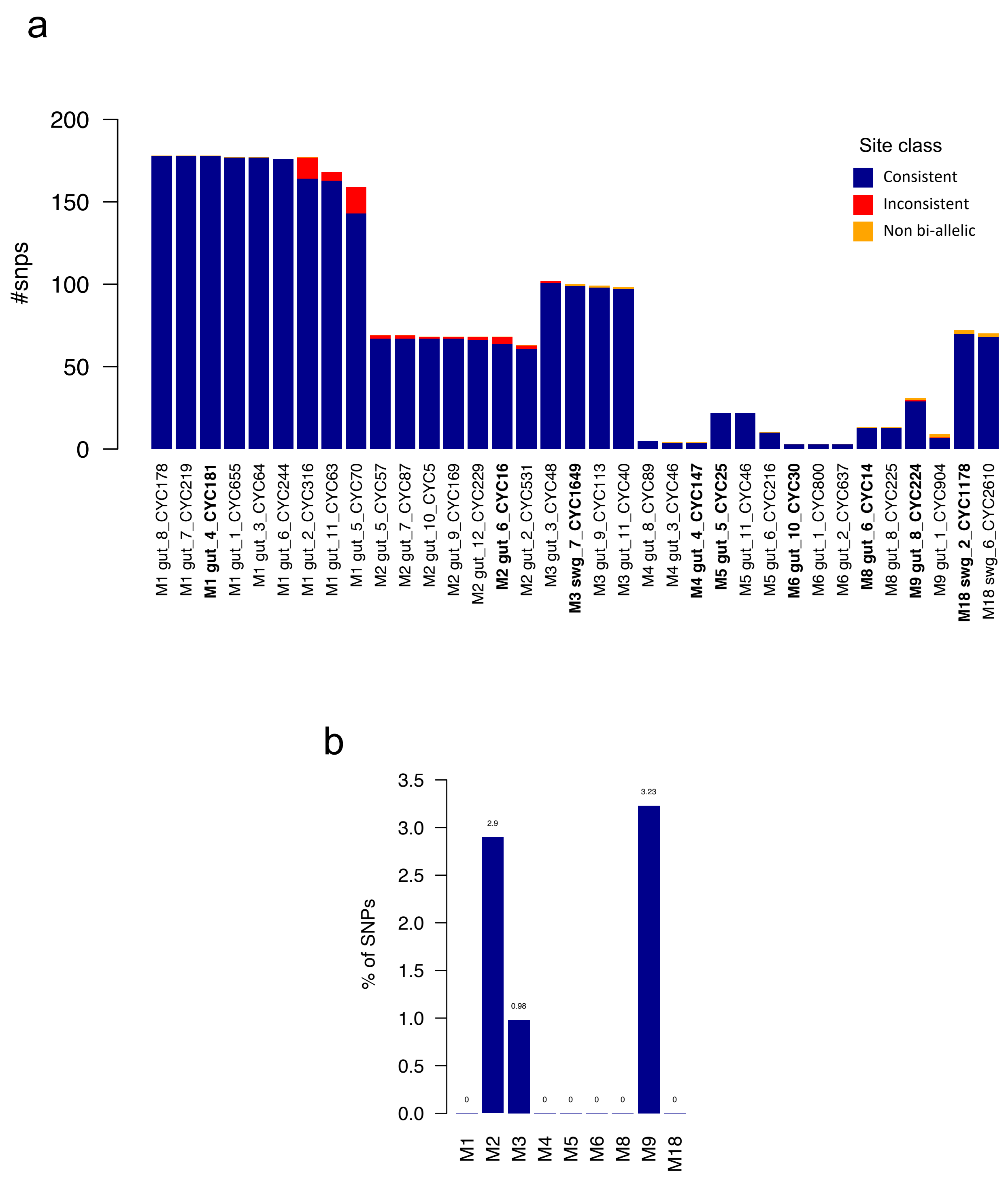


**Supplementary Figure 16.** Putative recombination events are rare. For each cluster and each cluster member, the phylogenetic tree (as inferred by PhyML) was used to classify all polymorphic sites. A bi-allelic site was classified as *consistent* if the partitioning of samples matched an edge in the tree, as *inconsistent* otherwise. Non bi-allelic sites were classified as such. **a)** The breakdown of site classification for all clusters and all options of pivot cluster members. The pivot members selected for visualization purposes in Figure 6 are highlighted in bold. **b)** The maximal percentage of consistent sites out of all sites over all cluster members. Clusters with a value of zero (such as M1) have at least one tree topology that is consistent with all polymorphic sites.

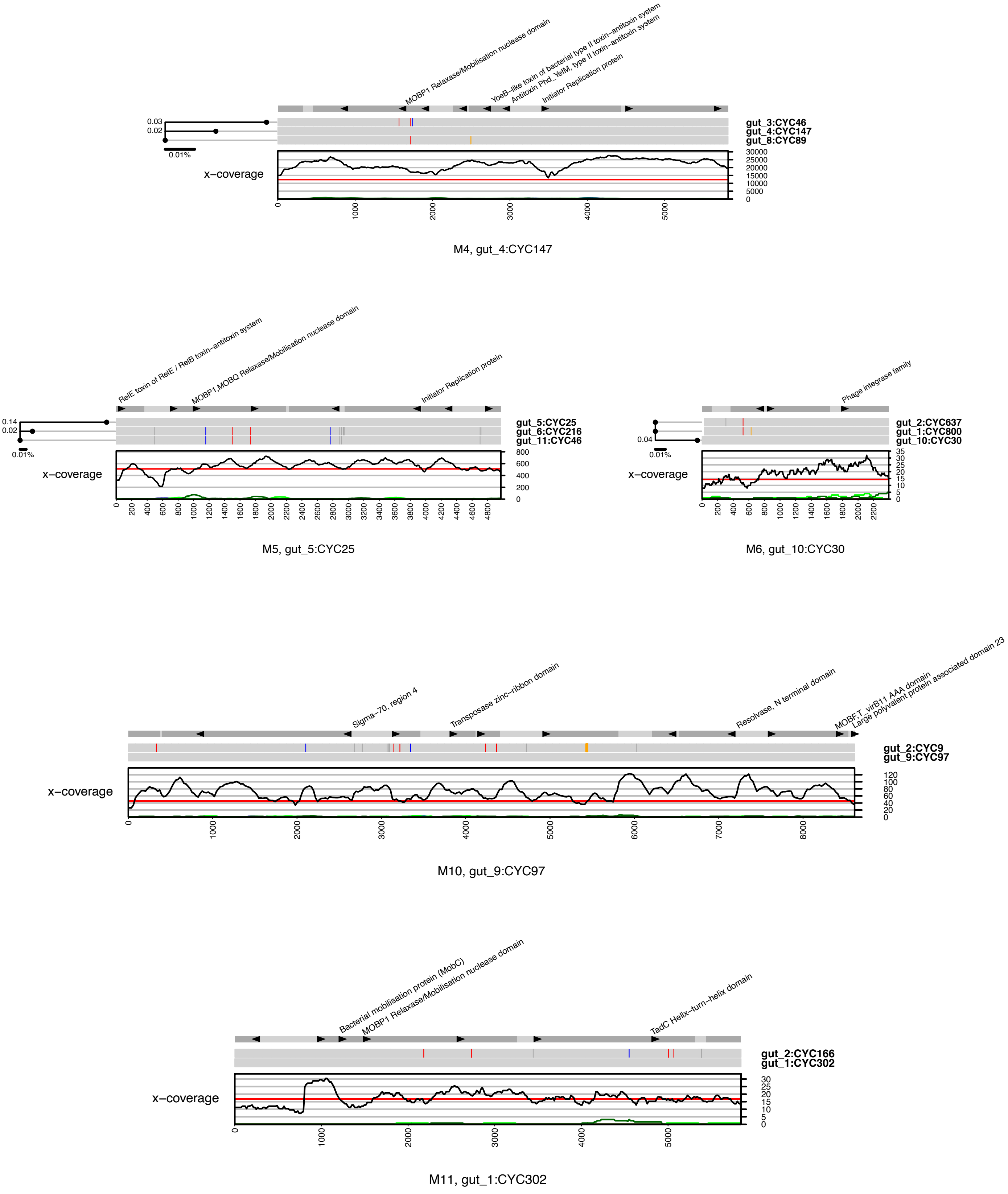


**Supplementary Figure 17.** Gut clusters with uneven distribution of SNPs along genomes. Groups of nearby SNPs can indicate a recombination event or positive selection. Legends as in Figure 6.
